# Multiscale Mechano-Biological Finite Element Modelling of Oncoplastic Breast Surgery—Numerical Study towards Surgical Planning and Cosmetic Outcome Prediction

**DOI:** 10.1371/journal.pone.0159766

**Published:** 2016-07-28

**Authors:** Vasileios Vavourakis, Bjoern Eiben, John H. Hipwell, Norman R. Williams, Mo Keshtgar, David J. Hawkes

**Affiliations:** 1 Centre for Medical Image Computing, Department of Medical Physics & Biomedical Engineering, University College London, Gower Street, London, WC1E 6BT, United Kingdom; 2 Division of Surgery & Interventional Science, University College London, 132 Hampstead Road, London, NW1 2BX, United Kingdom; 3 Department of Surgery, Royal Free Hospital, University College London, Pond Street, London, NW3 2QG, United Kingdom; Universidade da Coruna, SPAIN

## Abstract

Surgical treatment for early-stage breast carcinoma primarily necessitates breast conserving therapy (BCT), where the tumour is removed while preserving the breast shape. To date, there have been very few attempts to develop accurate and efficient computational tools that could be used in the clinical environment for pre-operative planning and oncoplastic breast surgery assessment. Moreover, from the breast cancer research perspective, there has been very little effort to model complex mechano-biological processes involved in wound healing. We address this by providing an integrated numerical framework that can simulate the therapeutic effects of BCT over the extended period of treatment and recovery. A validated, three-dimensional, multiscale finite element procedure that simulates breast tissue deformations and physiological wound healing is presented. In the proposed methodology, a partitioned, continuum-based mathematical model for tissue recovery and angiogenesis, and breast tissue deformation is considered. The effectiveness and accuracy of the proposed numerical scheme is illustrated through patient-specific representative examples. Wound repair and contraction numerical analyses of real MRI-derived breast geometries are investigated, and the final predictions of the breast shape are validated against post-operative follow-up optical surface scans from four patients. Mean (standard deviation) breast surface distance errors in millimetres of 3.1 (±3.1), 3.2 (±2.4), 2.8 (±2.7) and 4.1 (±3.3) were obtained, demonstrating the ability of the surgical simulation tool to predict, pre-operatively, the outcome of BCT to clinically useful accuracy.

## Introduction

Breast cancer is currently the most common cancer in Europe, with 464,000 cases recorded in 2012 [1]. It is also the most prevalent cancer (20%), surpassing both prostate (18%) and large bowel (13%) cancers. However, five year survival of breast cancer currently exceeds 80% in both Europe and the USA [2–4], with the result that more and more women are living with the consequences of their treatment. This treatment is primarily surgical, but may also involve adjuvant therapies, such as chemotherapy and radiotherapy. Whilst around 1% of breast cancer cases do occur in men, mastectomy is usually the recommended treatment. As this study is solely concerned with breast conserving therapy, male breast cancer is not considered further. Evaluation of the aesthetic outcome of breast conserving therapy suggests that 6% of patients receive a poor cosmetic result and up to 30% of patients may be dissatisfied with their appearance after treatment [5]. This has prompted interest in developing objective, standardised tools for the evaluation of the cosmetic outcome [6]. This outcome is a function of many factors, however, including tumour size and location, the volume of the breast, its density, and the dose and distribution of radiotherapy [5]. There is a clear requirement therefore, for tools that could predict the cosmetic outcome of breast surgery and support the collaborative decision making process between surgeon and patient in choosing from the range of surgical and therapeutic options available. These include identifying when simple breast conserving surgery may be viable, given shrinkage of the tumour following neo-adjuvant chemotherapy for instance, or whether more complex oncoplastic breast conserving surgery or mastectomy, with or without reconstruction, will be required in order to obtain the desired aesthetic outcome.

Wound healing is a complex biological process involving the coordinated actions of multiple cell types, a variety of important proteins and chemicals responsible for cell signalling, and micro-environmental conditions [7]. It is essential that surgeons understand the key physiological processes involved, such that the most important factors influencing successful wound healing can be identified and optimised. This could improve the outcome of breast conserving therapy (BCT) interventions and reduce negative effects from adverse wound healing.

An overview of the biological and molecular mechanisms that regulate wound healing and angiogenesis is provided in S1 File. A detailed understanding of these processes may enable new surgical techniques to be developed, enhance our ability to treat chronic wounds and improve our ability to deliver an optimal treatment for cancer. Mathematical modelling and computer simulations can further this aim by improving our understanding of the processes involved in tissue regeneration and vascular sprouting. The state-of-the-art in this field is outlined in the next two subsections.

### Mathematical Modelling of Tissue and Vascular Network Regeneration

Wound healing and angiogenesis are very challenging and complex mechano-biological processes to investigate and hence difficult to model mathematically. With the advent of bioengineering and high-performance computing, significant effort towards realistic and more accurate simulations of such biological processes has been carried out within the last two decades. In the literature various mathematical models that investigate certain aspects of soft tissue regeneration and vascular sprouting have been described. In this respect, the two-volume manuscript of Murray [8, 9] is an excellent resource in biology mathematical modelling, including tissue growth, dermal and epidermal wound healing, cancer development, etc.

Most of the existing mathematical formulations in the field are founded on the established Oster-Murray-Harris model [10] for morphogenesis. However, amongst the first to simulate epidermal wound recovery was Sherratt and Murray [11], who proposed a very simple mathematical model for the healing of circular epidermal wounds. Also, Tranquillo and Murray [12] employed a continuum model that couples the conservation laws for fibroblast cells and the extracellular matrix (ECM) density, with the balance equation for the epidermis. Olsen and colleagues [13] developed a more complex mechano-chemical model for adult mammalian skin wound contraction that also considers the dynamic consumption and production of the chemical stimulus in cell function and phenotypic transformation between fibroblasts and myofibroblasts. Ramtani et al. [14] presented a fibroblast-collagen-matrix contraction model to numerically study the biomechanical interactions that regulate wound contraction of connective tissue cells and investigated the effect of ECM remodelling in conjunction with ECM density, cell number and cell-matrix interaction stress.

Manoussaki was amongst the first to propose a coupled mechano-chemical model of angiogenesis and vasculogenesis using a finite difference scheme to simulate in-vitro vascular network formation [15]. A more detailed continuum-based model of angiogenesis was later proposed by Schugart et al. that consisted of a system of seven non-linear partial differential equations accounting the key components of wound healing [16]: capillary tips and sprouts, fibroblasts, inflammatory cells, a chemo-attractant, oxygen level and the ECM. Javierre et al. [17] extended the mathematical model of [13] by considering cell mechanical sensing and transmission of traction forces to the ECM, mechano-sensitive cell differentiation, and a dynamic change of the ECM mechanical properties with collagen deposition. Also, Xue and colleagues [18] presented a two-dimensional, mathematical model of ischemic dermal wound healing, where a set of coupled PDEs—involving the concentration of oxygen, vasculogenesis chemical agents, the density of macrophages, fibroblasts, capillaries and the density and velocity of the ECM—were solved monolithically. Flegg et al. [19] proposed a three-species (oxygen level, capillary tip and blood vessel density) mathematical model of wound healing to simulate the application of hyperbaric oxygen therapy in the treatment of chronic wounds. The model was later improved into a six-species one and was used to assess chronic diabetic wound treatment strategies with hyperbaric oxygen [20]. Valero et al. [21] presented a two-dimensional FE model for the simulation of skin wound recovery and scarring, where a mechano-sensitive biochemical model in angiogenesis is employed.

### Breast Tissue Biomechanical Modelling

Development of a high-fidelity patient-specific surgical simulator—namely a computational model of oncoplastic breast surgery (OBS) interventions—which could potentially be used in the clinical environment is a challenging task. Accurate and reliable biomechanical modelling requires knowledge of the anatomy and an understanding of the biology of the system under consideration. The female breast is composed of adipose tissue, glandular lobules and milk ducts, connective tissues and the dermis (see Chapter 2A in [22]). The glandular lobules and milk ducts are concerned with milk production and are surrounded by dense connective tissues, which are collectively referred to as fibroglandular tissue. The breast is attached posteriorly to the chest by the pectoralis fascia over the pectoral muscle, while its shape is established and maintained by the skin and the supporting Cooper’s ligaments.

In the past few years, novel computer algorithms have been developed in the field of breast cancer research, for surgical training and pre-operative planning, image guided surgery, diagnosis and clinical biopsy. Much of this work has focussed mainly on modelling the breast deformations associated with radiological acquisitions, such as mammographic plate compressions [23–27] for the purposes of image registration.

However, a small number of studies have been undertaken with a specifically surgical planning or outcome prediction focus. Roose et al. [28] developed a computer-based planning tool to predict the surgical outcome of breast augmentation procedures by sub-glandular placement of an implant above the pectoralis major. In related work, Lapuebla-Ferri et al. [29] presented an FE procedure of breast augmentation through silicone implant insertion. Del Palomar et al. [30] proposed a numerical procedure, based on the FE method, to simulate breast deformations after lumpectomy. Garbey et al. [31] presented a two-dimensional modelling approach to predict the surgery outcome after breast tumour resection, using a coupled FE biomechanical model and a cellular automata wound healing model. Patete et al. [32] developed and validated a three-dimensional female breast deformation model for computer assisted breast surgery and breast biopsy analysis, whose modelling approach was based on the mass-spring method.

This contribution presents the work of two EU-funded projects (see the Acknowledgments section) that aim to develop novel in-silico modelling tools of breast mechano-biology, and innovative techniques towards surgical planning and outcome prediction of BCT using a combination of various imaging modalities, i.e. magnetic resonance imaging (MRI), optical surface scans (3dMD), etc. In this paper we present a novel technique to biomechanically model the anatomy of the breast on a patient-specific basis. We propose an integrated predictive tool that accounts for the effect of surgical removal of early-stage breast carcinoma via lumpectomy; it predicts the post-operative results of wound healing, while it can potentially accommodate the side-effects of adjuvant chemo- and radiotherapy. Ultimately, the tool developed can be used to explore surgical strategies and the consequences of the available options with respect to the final appearance of the patient’s breast.

In this paper we deliver a three-dimensional surgical simulator for computer-aided surgical planning in BCT of early stage breast cancer patients. To date, there are no standard tools for aesthetic assessment and prediction of lumpectomy intervention outcomes, and no related tools to support patient-surgeon interaction or multi-disciplinary team meetings to aid a shared decision making process. To the best of our knowledge, this is the first attempt to model—in an integrative manner—breast tissue biomechanics and physiological soft-tissue recovery. More importantly, this work pioneers in presenting a validated modelling framework by using a combination of different imaging modalities (MRI and 3dMD surface acquisitions) before and 6 to 12 months after OBS.

## Materials and Methods

### Ethics

Patients gave written informed consent in line with Good Clinical Practice and the Declaration of Helsinki. The trial was approved by the London—Camden & Islington National Research Ethics Committee (reference 14/LO/1117) in accordance with the *PICTURE Breast L* (https://clinicaltrials.gov/ct2/show/NCT02341820) study protocol.

In this section, the finite element (FE) modelling procedure for oncoplastic breast surgery techniques, along with patient-specific biomechanical modelling is presented. The structure of this section is as follows: First, a description of the mathematical modelling of the wound healing and angiogenesis analysis is given, followed by an outline of the relevant biomechanical model of breast tissue. Subsequently, the coupled multiscale FE procedure for the developed mechano-biological modelling framework is described. This section concludes with the presentation of the onco-plastic surgical simulation tool.

### Wound Healing and Angiogenesis FE Model

The basic mathematical model for tissue recovery by Sherratt and Murray is followed here [11]. We adopt a five-species physiological wound healing and angiogenesis model that contains the following state variables: the cell density, *η*, the concentration of a biochemical agent that regulates mitosis, *ς*, the microvascular network density, *υ*, the oxygen and nutrient level, *ξ*, and an agent to regulate vascular sprouting, i.e. the macrophage-derived growth factor (MDGF), *μ*.

The cell density (e.g. fibroblasts, myofibroblasts, smooth muscle cells, etc.) balance is described by a reaction-diffusion partial differential equation (PDE), which in the general form is also referred to as the Kolmogorov-Petrovsky-Piskounov equation, i.e.

$$\begin{array}{c} \dot{\eta }=D_{\eta }\;\nabla^{2}\eta +k_{\varsigma }\;S\left(\varsigma \right)\;\left(2\;\eta_{0}\;\eta -\eta^{2}\right)-k\;\eta \;, \end{array}$$ (1)

where *D*_*η*_ is an isotropic diffusion coefficient (in m^2^/s), *η*_0_ is the reference cell density in the undamaged tissue region, and *k*_*ς*_ is a linear rate (given in m^3^cell^-1^s^-1^) that describes the production of cells in the wound, while *k* is the natural cell loss or death rate (in s^-1^). Following [11], function *S* reflects the chemical control of cell mitosis either as an activator or as an inhibitor, hence, taking the form respectively

$$\begin{array}{c} S\left(\varsigma \right)=\frac{2\;h_{1}\;\bar{\varsigma }\;\varsigma }{{\bar{\varsigma }}^{2}+\varsigma^{2}}+h_{2}\;,\;\;\text{and}\;\;S\left(\varsigma \right)=\frac{\left(h_{0}-1\right)\;\varsigma +h_{0}\;\varsigma_{0}}{2\left(h_{0}-1\right)\;\varsigma +\varsigma_{0}}\;, \end{array}$$

where *h*_0,1,2_ and $\bar{\varsigma }$ are parameters modulating the production rate of cells (see S1 Table).

In wound healing, chemical growth factors fulfil the task of triggering or suspending the production of soft tissue cells, i.e. breast tissue cells in this work. To date, various factors which chemically regulate cell mitosis and thus tissue recovery have been reported, two of which are the keratinocyte growth factor and the basic fibroblast growth factor. However, in the present mathematical model, a homogenised species *ς* is assumed to model biochemical regulation of cell division. Here chemical growth transport is described by the parabolic equation

$$\begin{array}{c} \dot{\varsigma }=D_{\varsigma }\;\nabla^{2}\varsigma +\ell_{\eta }\;\eta -\ell \;\varsigma \;, \end{array}$$ (2)

where *D*_*ς*_ is the corresponding chemical diffusion coefficient (in m^2^/s), while the second and third term on the right-hand side of Eq (2) describe the production of the chemical by the cells and the decay of *ς* respectively (see S1 Table).

Capillary-tip sprouting and endothelial wall formation can be mathematically modelled by a simplified version of Fisher’s equation where the diffusive term is omitted [33]:

$$\begin{array}{c} \dot{\upsilon }=\beta \;H\left(\mu \right)\;\left(\upsilon_{0}\;\upsilon -\upsilon^{2}\right)\;, \end{array}$$ (3)

where *υ*_0_ is the reference microvascular density under physiological conditions in the healthy tissue, defined as the ratio of capillary wall surface to tissue volume ratio (in m^-1^) [34]. Also, *β* is a proportionality parameter that represents the capillary growth rate (in m/s), and *H*(*μ*) = 3*μ*/(2*μ*_0_ + *μ*) is a function that relates the production of new capillaries in the wounded region with the concentration of chemicals promoting angiogenesis, i.e. the MDGF level, *μ*, (see Eq (5)).

Additionally, transport of oxygen and nutrients in the wounded-tissue space is mathematically modelled through the following reaction-diffusion PDE

$$\begin{array}{c} \dot{\xi }=D_{\xi }\;\nabla^{2}\xi +\lambda_{\upsilon }\;\upsilon -\lambda \;\xi \;, \end{array}$$ (4)

where *D*_*ξ*_ is the isotropic diffusion parameter (in m^2^/s), *λ*_*υ*_ the oxygen and nutrients production rate corresponding to the supply of the microvascular network (in m s^-1^), and *λ* the consumption rate of the species by macrophages and the regenerated tissues in the wound (in s^-1^). However, to render oxygen balance Eq (4) independent of the corresponding reference value of the oxygen/nutrient level, the state variable *ξ* is expressed above in dimensionless normalised form. Thus, we assume that normal blood oxygen level is considered when *ξ* ∈ [0.95, 1].

Macrophages, in addition to increasing inflammation and stimulating the immune system, also play an important anti-inflammatory role and encourage tissue repair. The latter, namely M2 macrophages, release growth chemicals usually referred to collectively as MDGF. MDGF in turn stimulates the proliferation of fibroblasts, smooth muscle cells and endothelial cells, as well as stimulating vessel growth and collagen remodelling [35, 36]. Following [33], MDGF-species conservation is modelled as

$$\begin{array}{c} \dot{\mu }=D_{\mu }\;\nabla^{2}\mu +\phi_{\xi }\;Q\left(\xi \right)-\phi \;\mu \;, \end{array}$$ (5)

where *D*_*μ*_ is the diffusion coefficient (in m^2^/s), while *ϕ* is the MDGF decay rate and *ϕ*_*ξ*_ the MDGF concentration rate under hypoxic conditions (given in s^-1^ and g cm^-3^s^-1^ respectively). The source function *Q* describes the dependence of the MDGF level with respect to the oxygen level in the wounded tissue site, and has the form:

Q(ξ)={1-ξξ^;ifwoundandξ<ξ^0;elsewhere,

where $\hat{\xi }$ represents the oxygen-level threshold below which macrophages release MDGF.

In order to effectively couple the balance equation that models cell transport with those that describe angiogenesis, we have modified the source term on the right-hand side of Eq (1) accordingly

$$\begin{array}{c} \dot{\eta }=D_{\eta }\;\nabla^{2}\eta +K\left(\xi \right)\;S\left(\varsigma \right)\;\left(2\;\eta_{0}\;\eta -\eta^{2}\right)-k\;\eta \;, \end{array}$$ (6)

where the production rate increases linearly when the oxygen and nutrient concentration is below a prescribed threshold $\tilde{\xi }$ and then is restored via the normal production value *k*:

K(ξ)={kςξξ˜;ifξ<ξ˜kς;elsewhere.

However, the above set of coupled equations can be represented in a more general matrix form

$$\begin{array}{c} \dot{q}=D\cdot \nabla^{2}q+S\left(q\right)\;, \end{array}$$ (7)

where **q** = {*η*, *ς*, *υ*, *ξ*, *μ* }^*T*^, **D** is a diagonal matrix whose components are the corresponding diffusion coefficients, and vector **S** contains the non-linear source or sink terms arising from Eqs (2)–(6).

The associated boundary and initial conditions to this boundary-value problem are as follows. The initial density of the cells and the microvasculature in the damaged tissue region is set to zero. Conversely, to obtain a stable mathematical physiological wound healing and angiogenesis model, it is assumed that cell density *η* = *η*_0_, oxygen level *ξ* = 1 and capillary density *υ* = *υ*_0_ in the healthy breast tissue region and the operated/healthy–interface boundary. The two biochemical growth-factor state variables, i.e. *ς* and *μ*, are initially set to zero everywhere. Additionally, the operated (damaged) tissue domain is assumed to be contained within the healthy (non-operated) one. Thus, the bounding surface of the computational domain of the analysed breast geometry is distant from the surface of the interface between damaged and healthy tissue. As a result, no boundary effects are expected to occur on the numerical results and, consequently, it is reasonable to prescribe zero-flux boundary conditions in the computational domain for the above biological and biochemical species.

The coupled reaction-diffusion Eq (7) is discretised using the finite element method, while time-integration is accomplished using an explicit numerical scheme. A brief description of the numerical solution procedure and the adopted parameter values are provided in S2 File and S1 Table respectively.

### Soft Tissue Biomechanics FE Model

Using quantities related to a reference configuration of the breast geometry, equilibrium of the soft biological tissues subjected to finite deformations can be described by the well-known Navier-Cauchy PDE [37]. The linear momentum equation in a Lagrangian frame of reference reads

$$\begin{array}{c} \nabla \cdot [F\cdot S]\;+\;\rho_{0,\text{tissue}}\;b\;=\;0\;, \end{array}$$ (8)

where **F** is the deformation gradient tensor and **S** is the symmetric 2nd Piola-Kirchhoff stress tensor; while *ρ*_0,tissue_ is the mass density of the breast tissue, and **b** the body force vector per unit of mass (e.g. specific gravitational force). However, as evident from Eq (8), inertia effects are ignored since in the present study the rate and magnitude of external loadings is very low.

In the present mechano-biological model, the stress distribution is calculated as the superposition of the corresponding passive stresses in the tissues owing to mechanical deformations and the active stresses effectively appearing in the wound during tissue recovery [17]: **S** = **S**_passive_ + **S**_active_. Assuming breast tissues (fibroglandular and adipose tissue) can be modelled biomechanically as an isotropic, quasi-incompressible, hyperelastic solid, then passive stresses can be computed through the constitutive relation: $S_{\text{passive}}=\partial {\bar{W}}_{\text{tissue}}/\partial E\;,$ where **E** is the Green-Lagrange strain tensor and ${\bar{W}}_{\text{tissue}}$ an objective scalar function (≥0) usually referred to as stored-energy function [37, 38]. In this work, biomechanical behaviour of breast tissues is described through a simplified Mooney-Rivlin constitutive law

W¯tissue=c1(I¯1-3)+c2(I¯2-3)+κ2(J-1)2, (9)

where *J* is the determinant of the deformation gradient tensor, and ${\bar{I}}_{1}$, ${\bar{I}}_{2}$ is the first and second invariant respectively of the deviatoric right Cauchy-Green tensor [38]. The parameter *κ* (in Pa) represents a penalty constant to enforce incompressibility of the tissues, which is equal to the bulk modulus in the small deformation regime. The values of the material parameters, in Eq (9), used in this study for both breast tissue types are given in S2 Table. In the current biomechanical breast model, the presence of skin tissue is also considered. The dermis and epidermis is approximated by an incompressible, non-linear, hyperelastic constitutive law that accounts for large deformations. An exponential function of the stored-energy potential, originally proposed by Veronda and Westmann, is adopted (see Eq 14 in [39]; *g* = 0)

$$\begin{array}{c} {\bar{W}}_{\text{skin}}=\alpha_{\text{skin}}\;[e^{\beta_{\text{skin}}\;\left({\tilde{I}}_{1}-3\right)}-1]+c_{2,\text{skin}}\;\left({\tilde{I}}_{2}-3\right)\;, \end{array}$$ (10)

The values of the material parameters *α*_skin_, *β*_skin_ and *c*_2,skin_ are provided in S2 Table, while a uniform 1.5 millimetre thickness of the skin is considered in this study.

It is widely known that fibroblasts play a major role in wound contraction (see [11, 40] and references therein), which are cells being chemotactically attracted to the wounded tissue. In this regard, fibroblasts generate traction forces as they advance through the ECM from the healthy to the damaged tissue region. As a simplified approach, cell traction is mathematically modelled as an isotropic stress tensor, with the 2nd Piola-Kirchhoff active stress distribution expressed by: **S**_active_ = *τ*_f_ *η*_f_ **I**, where **I** is the identity matrix, *τ*_f_ the specific traction force exerted by the fibroblasts onto the collagen fibres in the ECM, and *η*_f_ is the fibroblast cell density in the wounded region. This model assumes *η*_f_ to be proportional to the cell density parameter: *η*_f_ = (*α*_f_ *η*/*η*_0_)*η*_0,f_, where *η*_0,f_ is the fibroblast cell density in the undamaged tissue region and *α*_f_ a dimensionless proportionality parameter. Additionally, the present formulation assumes a non-mechano-sensing model; hence, the above constitutive relationship is independent of the specific deformation, as opposed to the active mechano-sensing models of Manousaki [15] and Moreo et al. [41].

Another important aspect of this wound healing model is the mathematical description of tissue recovery. In this regard, a damage factor is introduced, *ζ*, also referred to as the reduction factor (see Chapter 6 in [38]). This internal scalar variable takes values within the range: *ζ* ∈ [0, 1) and we call it “damage variable.” The damage variable quantifies the magnitude of isotropic damage in the continuous hyperelastic solid and describes the health condition of the material, i.e. the soft tissue. In the present numerical methodology, the damage variable is assumed to be inversely analogous to the cell density in the operated tissue region, i.e. *ζ* = 1 − *η*/*η*_0_. At the beginning of the wound healing simulation, *ζ* ≈ 1 inside the wound (since *η* ≈ 0) and zero elsewhere (since *η* = *η*_0_). However, during the course of the analysis the damaged region recovers, hence *η* increases and therefore the structural integrity of the extracellular space, i.e. the tissue connectivity and coherence, increases in the same fashion. Thus, we scale the dilational part of the strain-energy function in Eq (9) of the damaged tissues by the integrity factor, (1 − *ζ*). The volumetric part of the tissue biomechanical constitutive equation is left intact in order to effectively model the quasi-incompressible behaviour of the fibrotic tissue in the wound due to the presence of biological fluids (e.g. blood plasma, serous fluid, blood clot). Additionally, *ζ* is set to zero throughout the analysis for the non-operated (undamaged) soft tissues of the breast model.

The elliptic Eq (8) is discretised using the finite element method. Moreover, the inertia contribution in Eq (8) has been ignored, since the time evolution of the healing process is slow, and rate effects are not considered in the present biomechanical model. Consequently, a quasi-static, fully-implicit Total Lagrangian FE formulation [42] is implemented, where the presence of material and geometric non-linearities in the elastic continuous medium are accounted for in the analysis. Additionally, the material constraint of biological soft tissues (i.e. near-incompressible behaviour) necessitates special formulation considerations in order to avoid degradation of the numerical results. To achieve this, hourglassing and volumetric locking side-effects are effectively avoided by using a mixed *u/p*-finite element formulation, originally proposed by Sussman and Bathe [43]. In this approach, hydrostatic pressure is introduced as an extra unknown in the system and is separately interpolated from the unknown displacement field variables. Following the previous section, the computational domain consists of two non-overlapping, interfacing volumes: the wounded tissue and the undamaged tissue surrounding the wound. As it will become evident in the following section, the initial values for the displacement and pressure variables are set to zero everywhere. The corresponding boundary conditions of the biomechanical boundary-value problem are traction-free for the skin surface, fixed displacements at the chest interface between the breast and the pectoralis fascia and prescribed displacement components within the clipped planes of the analysed breast domain (see the Results section for details).

To summarize the above, a three-dimensional, nonlinear FE methodology to solve the linear momentum equations for soft tissue biomechanics is employed. A detailed description of the numerical formulation and FE discretisation can be found in the references cited above.

### Multiscale FE Model

**Fig 1:**
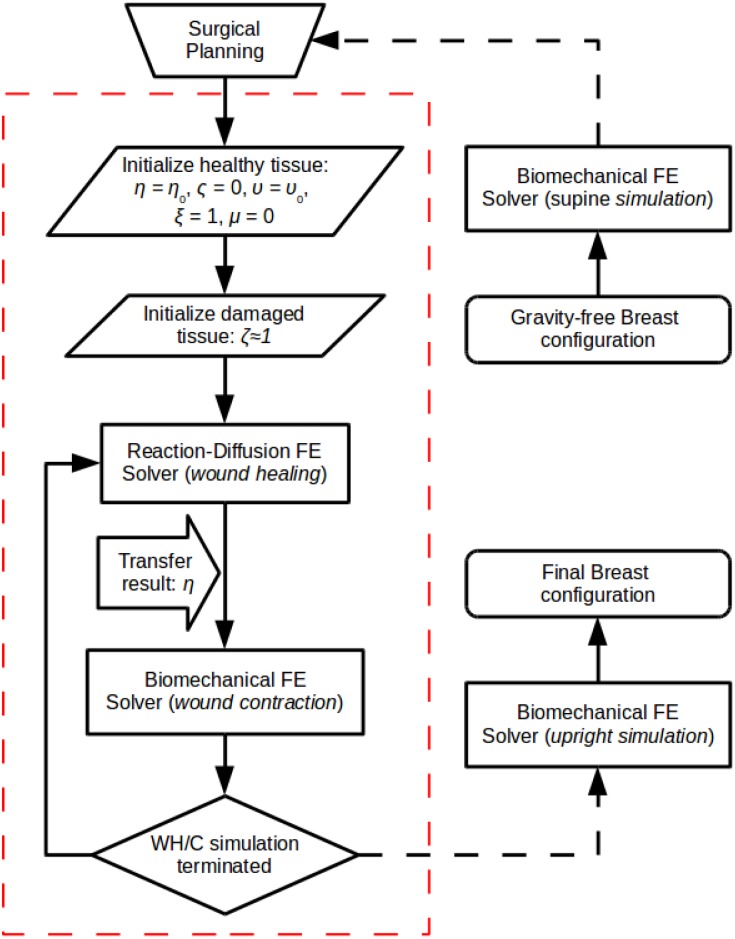
Flowchart of the multiscale FE solver. The red-dash line frames the mechano-biological wound healing, angiogenesis and wound contraction numerical methodology (where WH/C is wound healing/contraction).

The proposed multiscale, mechano-biological FE procedure has been implemented in C++ and incorporated into our in-house numerical analysis framework *FEB3* (see S3 File for details). Fig 1 illustrates a flow-diagram of the 3D multiscale FE angiogenesis, wound healing and contraction numerical procedure (red dashed frame). The multiscale simulation starts by considering some reference configuration (with an initial stress distribution field) of the analysed breast geometry and a user-defined surgical plan that identifies the undamaged and the damaged breast tissue regions (see subsection: Surgical Simulator for further details). The reader should note that the associated boundary and initial conditions of both (i.e. the wound healing and the biomechanical) FE models are as described in the above subsections.

The developed FE solver involves the numerical solution of the coupled non-linear reaction-diffusion equations (wound healing and angiogenesis FE solver) and the linear momentum equations (tissue solid mechanics FE solver) in a partitioned approach. The nature of multiscaling in this formulation is due to the different time scales at which the biochemical and the biomechanical analysis occurs. More specifically, time-integration of the transport equations for the wound healing angiogenesis simulation is accomplished through an explicit scheme, where the order of the time increment is in the order of seconds. In contrast, the pseudo-time increment for the biomechanics solver is of the order of several hours. In all surgical simulations in the present study, the adopted time steps for the two FE solvers are specified as 17.28 seconds and 6 days, respectively. Evidently, the time-increment for the reaction-diffusion solver is set very small in order to satisfy the Courant-Friedrichs-Lewy condition due to the stability limitation of the adopted time-integration scheme. Nonetheless, the biomechanical solver assumes that breast tissues deform quasi-statically hence the coarse time resolution. Also, in the proposed numerical procedure, the state variable, *η*, which describes the cell density in the wounded region is shared between the two FE solvers, as shown in Fig 1. Thus, this quantity effectively couples the biochemical and biomechanical parts of the model.

Moreover, since all governing equations are expressed in a fixed frame of reference, i.e. the Lagrangian frame, the analysed domain remains unchanged throughout the transient analysis, hence no FE-mesh updates and no mapping of the state variables is required.

To simulate the full tissue recovery process, the two analysis parts are alternated several times up to the desired period of simulation time. In this work, a three month period of healing is considered, where under physiological conditions the proliferative phase has been naturally completed and scar maturation takes place through the last stage of remodelling and scar formation. However, the proposed numerical formulation can be readily extended to an active mechano-sensitive wound contraction and angiogenesis mathematical model, similar to the models proposed by Olsen at al. [13] and Javierre and colleagues [17, 21]. In the present work, we considered a simplified mechano-biological model of wound healing and angiogenesis that requires an adequate—but not excessive—number of material parameters to describe the mechano-chemical model. Additionally, the software architecture of the present FE solver provides the option to modify the present healing and angiogenesis model from physiological to pathogenic conditions. This can allow us to further enhance this numerical framework and include radiotherapy or chronic disease informed biomechanical models for soft tissues.

The two core modules of the three-dimensional FE code, namely the reaction-diffusion explicit solver and the non-linear implicit solid solver, have been tested and verified separately by simulating simple benchmark tests—e.g. healing of epidermal wounds as in Valero et al. [21], or large deformation simulations of breast tissues as in Han et al. [26]—and the accuracy of the results has been successfully compared with the commercial software *ABAQUS*.

### Surgical Simulator

We have developed an integrated software platform—referred to as the *surgical simulator*—the core of which is the mechano-biological finite element analysis solver. The simulator includes the essential numerical algorithms that link the solver with the various input and output stages of the work-flow, such as the pre-processing of patient-specific imaging data, and providing the final output: the oncoplastic breast surgery simulation.

**Fig 2:**
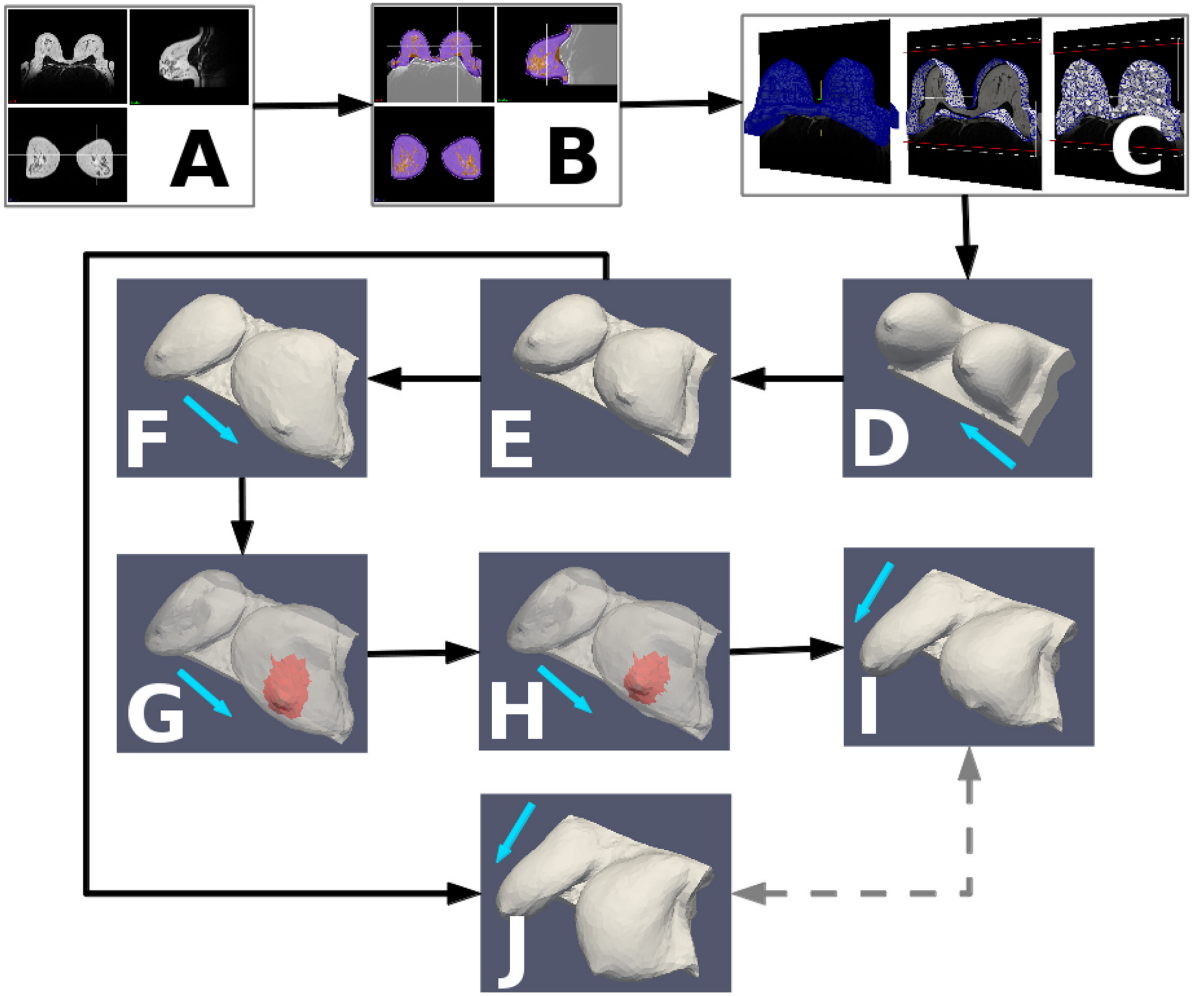
Pipeline of the computational procedure of the surgical simulator. The prone MRI (A) is segmented into tissue types (B) and a labelled volumetric mesh is created, which forms the basis of the biomechanical model (C). From the prone configuration of the model (D), the unloaded state is computed (E), from which re-application of gravity simulates the supine (F) and upright (J) configurations prior to surgery. Surgical planning, to specify the resected surgical “wound” (shown in red), is performed in the supine pose (G) and results in a cosmetic outcome prediction (H) which is transformed into the upright configuration (I) for presentation to the user. The pre- (J) and post- (I) surgical simulations can then be compared (dashed arrows) to reveal the predicted deformation of the breast due to surgery. The blue arrows indicate the direction of gravity: (D) from posterior to anterior (i.e. prone), (F,G,H) from anterior to posterior (i.e. supine) and (I,J) superior to inferior (i.e. upright). Note in G and H, the breast skin is shown transparent to visualise the excision region located inside the breast model (see also the adopted surgical plans in the Results section).

Fig 2 illustrates the work-flow of the proposed computational procedure of the three-dimensional multiscale FE surgical simulator. The process begins with step A, where MRI or computed-tomography (CT) scans are acquired. If only three-dimensional surface scans are available (i.e. no MRI or CT), then—for the reasons explained below—this and the following step is skipped.

In step B, the patient-specific imaging data are segmented. In this procedure the breast is delineated from the background and internal structures (i.e. adipose and fibroglandular tissues) are differentiated. Subsequently, two- and three-dimensional patient-specific meshes are generated in step C, while in step D, the model is prepared for input into the FE solver. In this regard, we employed the open-source software *Gmsh* (http://geuz.org/gmsh/) to generate high quality three-dimensional finite element meshes. More details about pre-processing steps B to D can be found in the published works by the authors [44, 45].

Step E involves the computation of the unloaded (i.e. gravity-free) configuration of the patient-specific breast geometry. Typically, breast MRI acquisition takes place with the patient prone, in which the breast is pendulous and extended anteriorly due to gravity. As a result, the image derived patient-specific breast geometry, and hence the state of the biomechanical model, is initially situated in a loaded condition (or under mechanical stress). We therefore carried out an inverse problem analysis to retrieve the unloaded (or gravity-free) state of the breast tissues [46]. Once the inverse simulation is finished, the FE mesh is updated by incrementing the nodal coordinates with the newly computed displacement vector field, thus, transforming the discretised breast geometry into the reference (or undeformed) setting.

Having virtually removed the gravitational load in the FE model, the corresponding supine or upright shape of the breast geometry can be predicted in steps F and J respectively. This is performed through conventional forward FE analysis simulations, as described in the Soft Tissue Biomechanics FE Model subsection. In these analyses the breast tissue deformation is assumed to be solely due to gravity, hence giving a prediction of the pre-operative patient-specific breast shape in the supine or upright configurations. The corresponding supine simulation is, in step F, used to represent the configuration of the breast in the virtual operating room.

In step G, virtual surgery takes place in which the surgeon identifies the tumour position in the digital breast cancer patient, and then makes a decision about which surgical plan to adopt. In this regard, the surgeon identifies the incision lines, the outline of the excised tissues, etc. on the discretised, three-dimensionally rendered breast model in the operating setting (i.e. in supine pose). Subsequently, the input data of the FE model are automatically updated accordingly, where the “resected” finite elements are labelled as damaged tissue (illustrated in red in Fig 2G). The multiscale wound healing simulation is performed in step H, as described in the Multiscale FE Model subsection. Naturally at the end of the simulation of step H, the wound will contract, resulting in an estimate of the new breast shape several weeks after BCT treatment.

Finally, in step I, the direction of gravity is re-applied in order to predict the follow-up breast shape in the upright position. Last but not least, the computed pre-operative upright breast geometry, step J, and the corresponding post-operative prediction, step I, can be subsequently used by the surgeon (and the multi-disciplinary team) to assess the virtual OBS technique and, if necessary, investigate alternative surgical strategies.

## Results

In this section, BCT intervention simulations are presented. The numerical investigation study involves patient-specific MRI data of four breast cancer patients. These patients were recruited at the Royal Free Hospital in London, in accordance with the *PICTURE Breast L* protocol and patient consent was obtained (see Ethics). All acquired clinical data were anonymised and, to allow internal processing, the following codes *P-1*, *P-2*, *P-3* and *P-4* are associated with the data-set of each patient respectively. None of these patients received adjuvant or neo-adjuvant chemotherapy but all received post-surgical radiotherapy.

Orthogonal views of the MRIs of the four patients considered in this analysis are shown in S2 Fig. The images are structural, non-contrast-enhanced, non-fat suppressed, T1-weighted (*P-1* and *P-4*), or where available T2-weighted (*P-2* and *P-3*). The spatial resolution of the MRI sequences is between 0.57 × 0.57 × 3 mm^3^ and 0.71 × 0.71 × 3 mm^3^. Both sequences allow good spatial differentiation between adipose and fibroglandular tissue and do not require different processing during the generation of the biomechanical model. As mentioned above, image acquisition is carried out with the patient lying in the prone position and the breast is pendulous in a dedicated receiver coil with cushioned patient support. Therefore, due to the effect of gravity, the breasts are naturally pulled into the anterior direction.

Careful inspection of the images and 3D models reveals an important limitation imposed by the MRI acquisitions: the skin of the patient is frequently in contact with the scanning device. This contact is especially apparent for patients with larger breast sizes, such as *P-1*, *P-3* and *P-4* (see S2 Fig) but it is also present for patient *P-2*. Hence, gravity is not the only load deforming the breast tissues and the skin, but also surface traction forces are present due to the rigidity of the support system and friction between the surfaces involved. Presently, it is not experimentally feasible to quantitatively measure those contact forces during imaging. As a consequence, during the FE model preparation for the surgical simulation (see step D in Fig 2) contact and friction have been ignored. Inevitably, it is expected that the inverse simulation in step E could potentially give an erroneous estimate of the gravity-free configuration of the analysed patient-specific breast geometry.

**Fig 3:**
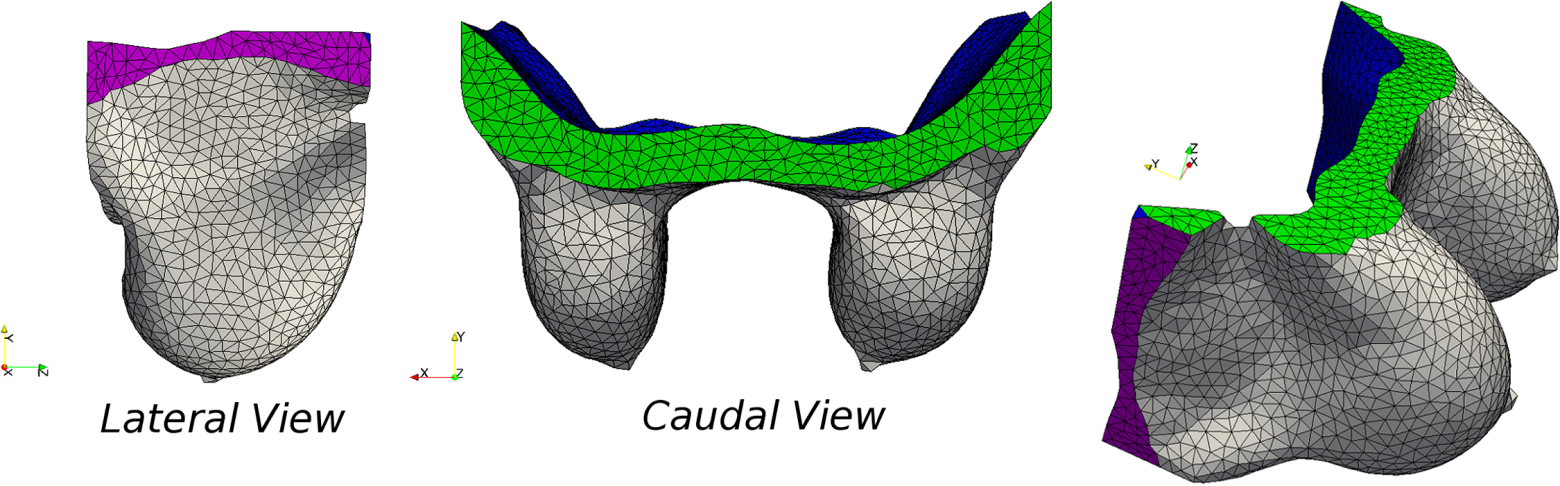
FE mesh and boundary conditions on the patient-specific breast model. Different views of the 3D finite element unstructured mesh of the *P-1*-specific breast model in prone position. Each colour represents the boundary condition considered in the model: traction-free skin and lateral surface, Z-component of displacement is zero prescribed on the superior and inferior horizontal (Y-plane) boundaries, and chest-wall is fixed.

The FE models (see illustrations in S3 Fig) are generated following the procedure described in the Surgical Simulator, starting with segmentation of the MRI scans of the patients. The analysed domain of the FE models is bounded by clipping the segmented breast image in the superior and inferior aspects as well as laterally (see Fig 3). Subsequently, three-dimensional finite element unstructured meshes of all patient-specific models are generated. The meshes comprise of 5,235, 4,213, 4,571 and 5,110 nodes, having 21,556, 15,485, 19,162 and 21,100 tetrahedral elements respectively.

In addition, boundary conditions are applied to the models in order to resemble anatomical conditions (see Fig 3). The skin and the two lateral clipping planes are considered traction-free, the displacements on the pectoralis major fascia interface are fixed completely (i.e. *u*_X,Y,Z_ = 0), while the axial displacement is fixed (*u*_Z_ = 0) in the horizontally clipped planes. The initial conditions for the boundary-value problem of the coupled mechano-biological mathematical model are described in the Wound Healing and Angiogenesis FE Model and Soft Tissue Biomechanics FE Model subsections.

**Fig 4:**
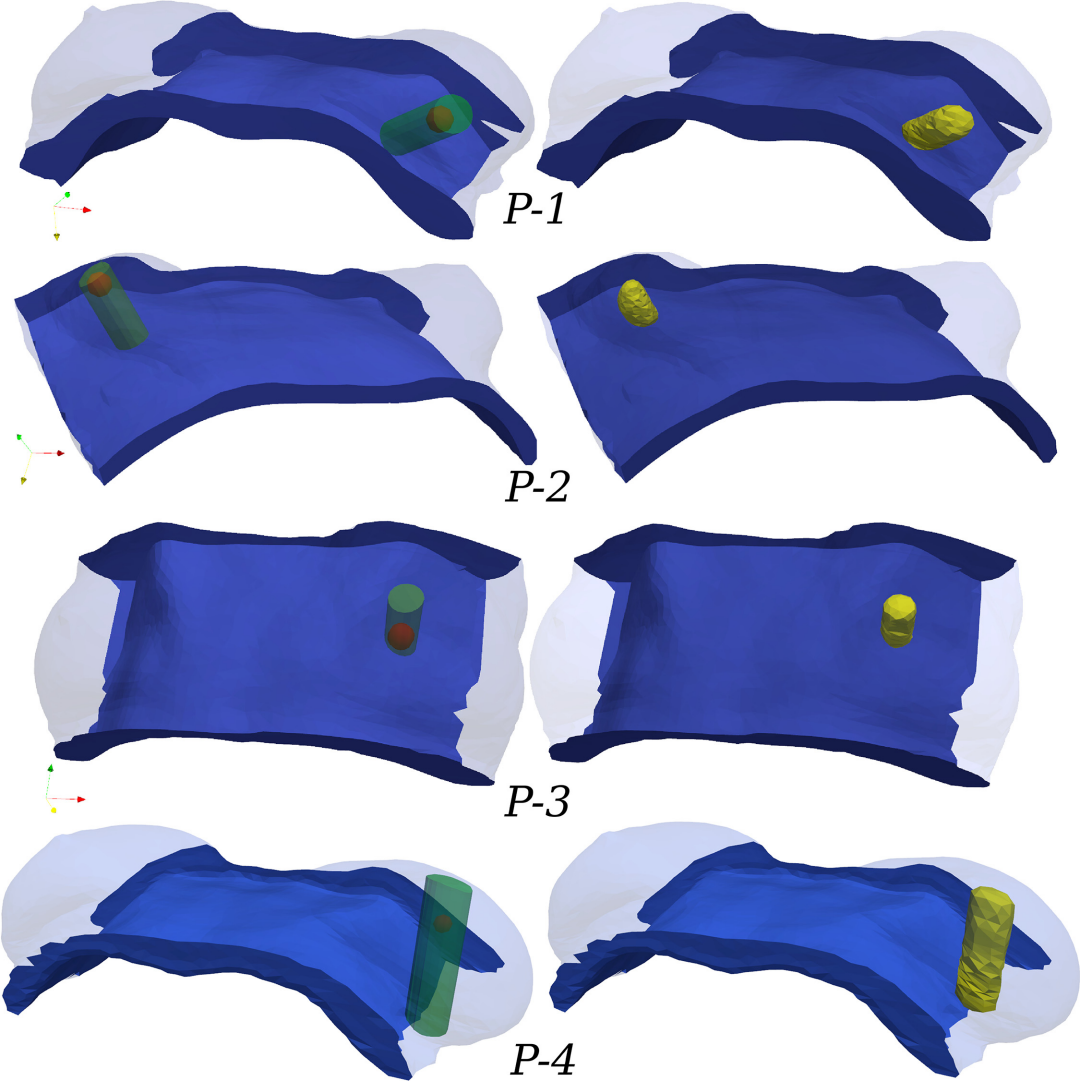
Adopted surgical plans in supine configuration, depicting the tumour location and the breast tissue resected. The “idealised” surgical plan (SP) for each patient (recorded by the breast surgeon) is presented in the left column. Tumour position is shown with a red sphere and the excised breast tissues with a green transparent cylinder. In the right column, the FE models are informed by labelling the corresponding volume elements as resected tissue (shown in yellow).

Virtual surgical planning is ideally done in the same orientation as the patient in the operating room environment, which is usually the supine position. As such, Fig 4 illustrates all patient-specific breast geometries in the simulated supine pose, where the tumour is shown as a spheroid (left column). In the same figures, the surgeon has defined the volume of the excised tissue including margins, has drawn the incision lines on the skin and the incision path inside the breast (not shown here) which is not directly related with the excised tissue. In the present study of in-silico breast surgery, we adopted a rather conventional approach of tissue resection during OBS. Breast tissue and lesion removal is accomplished by considering a cylinder, whose axis is perpendicular to the chest-wall surface and extends from the skin to the fascia. Tissues enclosed by the cylinder are expected to be removed by the surgeon in real surgery, and hence are “virtually excised” from the patient-specific model. However, we assume that no skin is resected or severely wounded, while the operated breast tissue is encapsulated by a reasonably smooth boundary that does not intersect with the fascia or the skin. Thus, the FE model is updated accordingly in order to differentiate the non-operated part of the breast tissues from the excised tissue region. In this regard, the tetrahedral elements of the FE volume mesh that lie inside the operated region are marked as wound (as in the segmentation step; see Fig 2B) having different biomechanical properties from the healthy counterpart (see S2 Table). Fig 4 (right column) depicts the tetrahedral elements (in yellow) of the volume mesh that correspond to the wounded site. Also, S3 Table summarizes some characteristics of the four patient-specific breast models. These include the total volume of the analysed domain of the breast, the volume of the excised tissues, and the amount of adipose and fibroglandular tissues. It becomes evident from this table that patient-specific breast model *P-1* represents a relatively fatty breast (having a fibroglandular-to-adipose tissue ratio of approximately 1:6) as opposed to patient *P-2* which can be regarded as very dense (having a 1:3 ratio). Note that the volume ratios are calculated over the breast region only. In contrast, the simulated domain extends further lateral and posterior to clipping boundaries that were set to be reasonably far away from the breast in order to minimize effects from the boundary conditions on the simulation. Hence the whole simulated domain is bigger than what is normally regarded as the anatomical definition of the breast as an organ.

Skin is also considered in the present study and is modelled using membrane elements. The dermis with the epidermis is assumed to be uniform in all breast models, 1.5 mm. Given the thickness and skin surface area, the total mass of the skin for each patient-specific model is estimated to be 162 g, 92 g, 187 g, and 220 g respectively.

The material parameters used in the present analysis for the biochemical and biomechanical models are provided in S1 and S2 Tables. A large number of parameters are taken from the literature, whereas some parameters (especially those for the angiogenesis biochemical model) have been estimated after numerical trials when plausible results are obtained.

**Fig 5:**
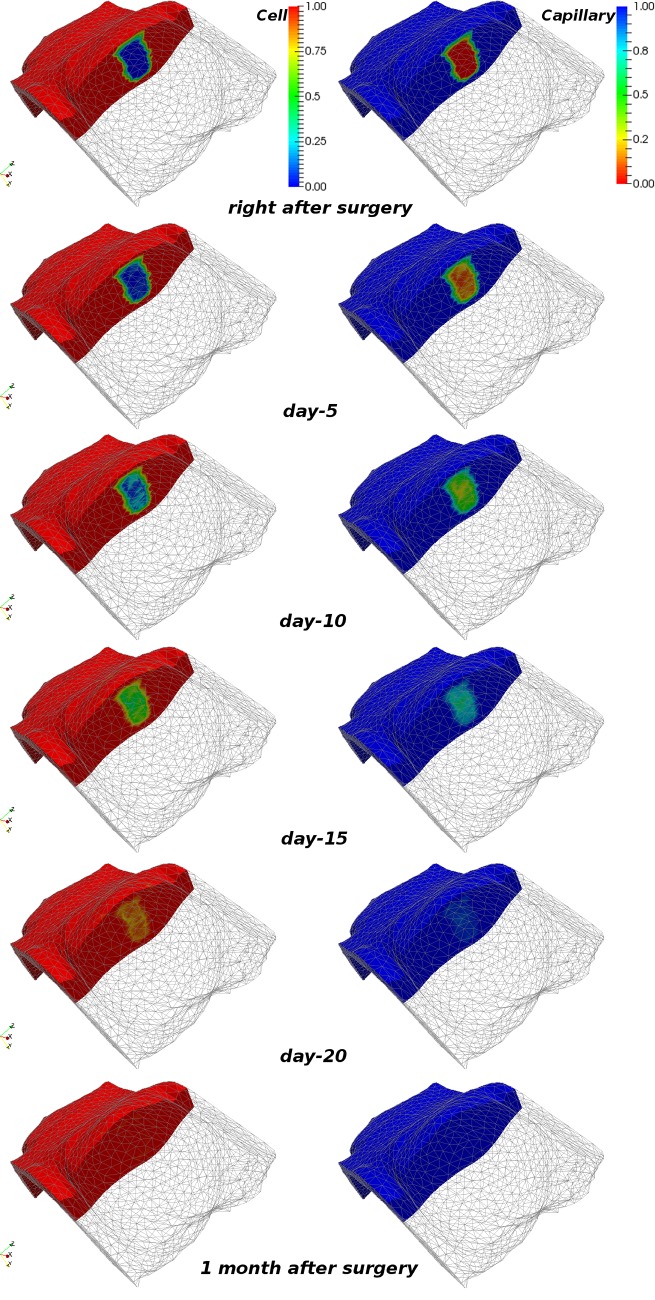
Snapshots of the cell and capillary density predictions during wound healing simulation. Numerical results of the wound healing simulation for patient-specific model *P-3* (in supine) depicting the evolution of cell and capillary density at various time instants. The operated breast is clipped to facilitate visualisation of the dynamic changes of the species in the wounded area. The left and right column depict the (normalised dimensionless) cell and capillary density, respectively.

Fig 5 shows the normalised dimensionless cell density, *η*, and capillary density, *υ*, at various time frames during the wound healing process for case *P-3*. Both quantities initially have zero value in the damaged region. During the course of the simulation, the healing mechanism induces tissue repair and the species at the end of the healing process reach balance. The terminal value of the species *η*, *υ* and *ξ* (not shown here) in the now-recovered tissue region is equal to *η*_0_, *υ*_0_ and 1 respectively, as in the non-operated breast tissues. We have also carried out simulations (not presented herein) where we tested both chemically-driven cell mitosis mechanisms (as described in the Wound Healing and Angiogenesis FE Model subsection) i.e. activator and inhibitor [11]. Interestingly, and in agreement with the results reported by Sherratt and Murray, we observed that biochemical inhibition and activation of mitosis produced very similar numerical results in terms of the soft tissues wound recovery rate, and the breast skin deformations. However, the present study considered the activator mitosis mechanism in the wound healing simulations.

**Fig 6:**
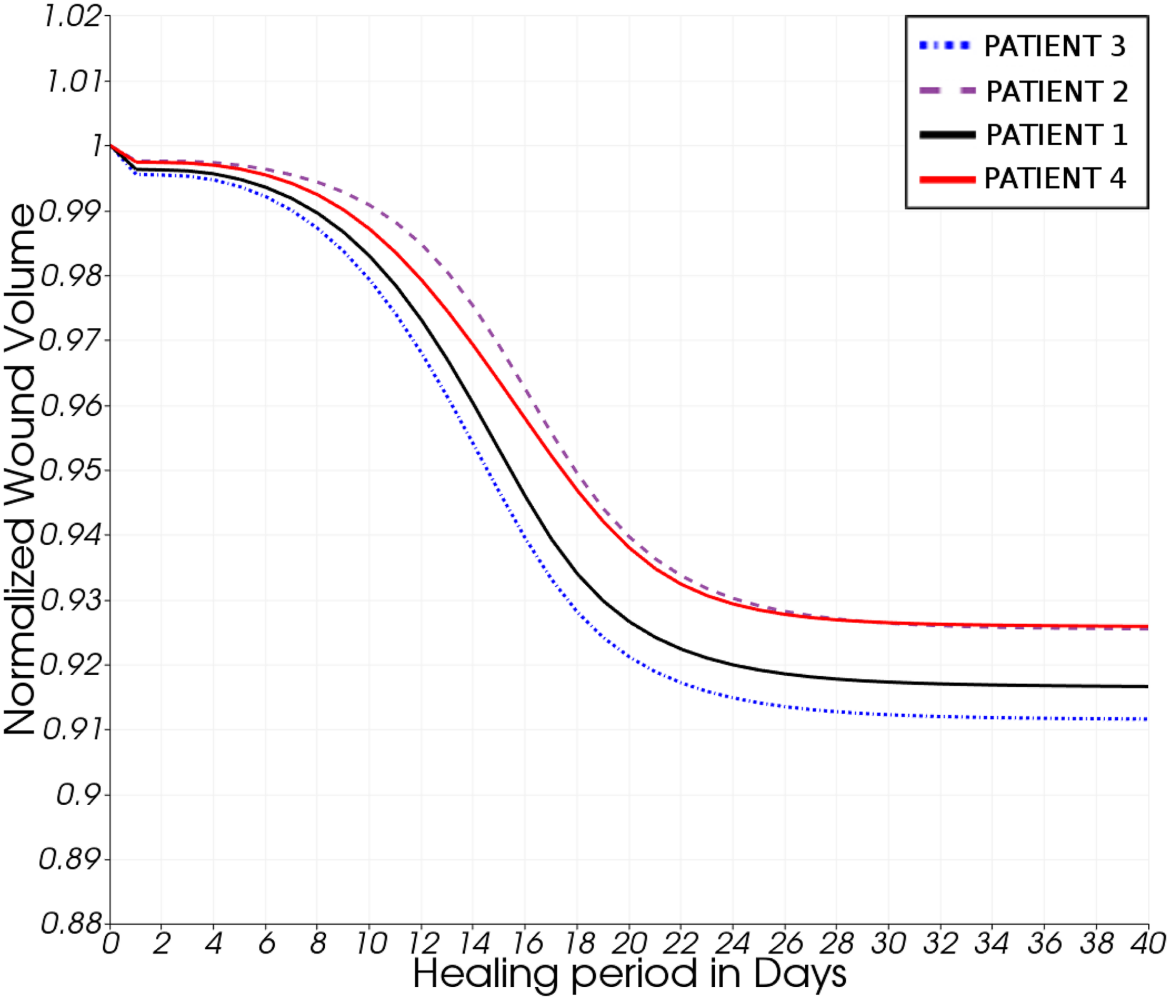
Predicted volume reduction of the operated tissue region during wound recovery. Numerical results of the normalised volume reduction of the operated (excised) tissue region during wound healing of all four patient-specific models. Note the initial slow volume contraction due to inflammation, followed by a pronounced contraction during the proliferative stage (day-8 to day-16) and final convergence to a smooth plateau in the remodelling phase (day-24 and onwards) of healing.

The original shape of the wounded region is not preserved during healing, and contraction is observed to be more pronounced at the low-curvature regions of the wound surface (see animation in S1 Video). These predictions are qualitatively in agreement with the wound contraction observations reported in the previously published mathematical models of epidermal tissue scarring [13, 17]. Fig 6 illustrates the relative wound contraction of all example cases over the course of the simulation time. The contraction to between 91 and 93% of the original volume is, of course, largely dependent on the choice of biochemical parameters (see S1 Table). The same holds true for the temporal course of the contraction. With the current set of parameters it is suggested that the breast reaches its final shape after about 30 days. Note that this simulation assumes no further treatment after surgery, which is of course a simplification of the post-surgical patient work-up. Regarding wound contraction, it has been observed by clinicians that the breast tissues at the wound site (after lumpectomy) stiffen as a result of the healing process and fibrosis occurring in the vicinity of the wound. This observation is supported by the reported possible disfigurements caused by BCT, and the proposed breast model takes this effect into consideration.

**Fig 7:**
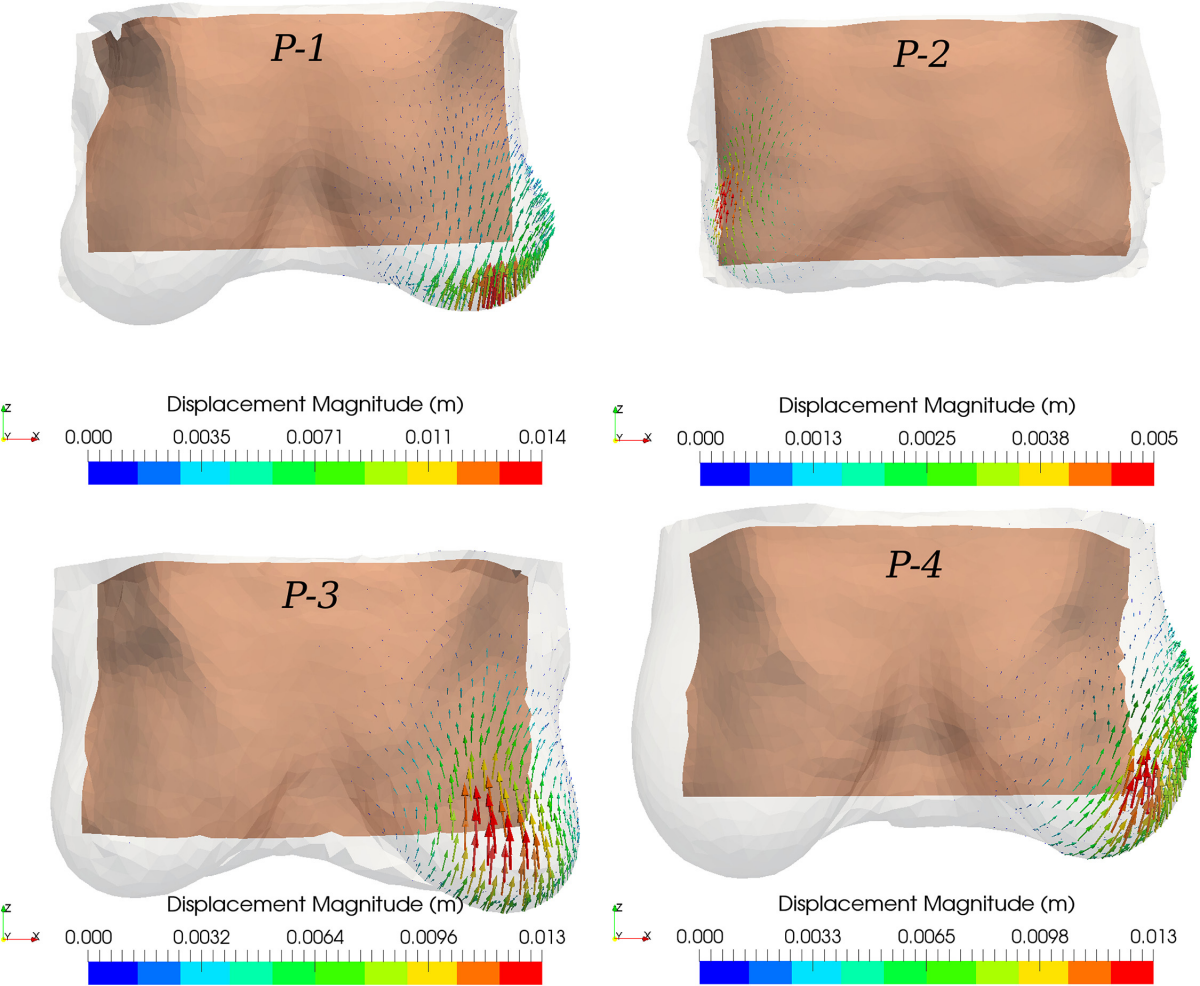
Vector representation of breast surface deformation after BCT. Arrow vector representation of the computed displacement field on the breast surface of the patient-specific post-operative model (after wound healing) with respect to the pre-operative setting in the upright position (transparent grey surface). The tan-coloured background surface corresponds to the chest wall boundary of the in-silico model. Note that all breast geometries are shown in the same scale.

The simulated wound contraction results in a deformation of the breast which, due to the connectivity of breast tissues, has an effect on the surrounding tissues. As shown in the surgical plans above (see Fig 4), the excision volume for patient *P-1* is located in the lower outer quadrant, for patients *P-2* and *P-4* in the upper outer quadrant, and for patient *P-3* in the upper inner quadrant. Thus, depending on the size and location of excised tissue, the maximum displacement magnitude in the upright position ranged from 5 mm (patient *P-2*) to 1.6 cm (patient *P-4*) approximately. The vector illustrations in Fig 7 highlight the deformation distribution on the breast skin (magnitude of the displacement vector in metres). As evident from this figure, after BCT treatment and recovery, tissue deformation is more pronounced at the breast skin surface proximal to the wounded region, which then causes the overall breast shape to deform.

**Fig 8:**
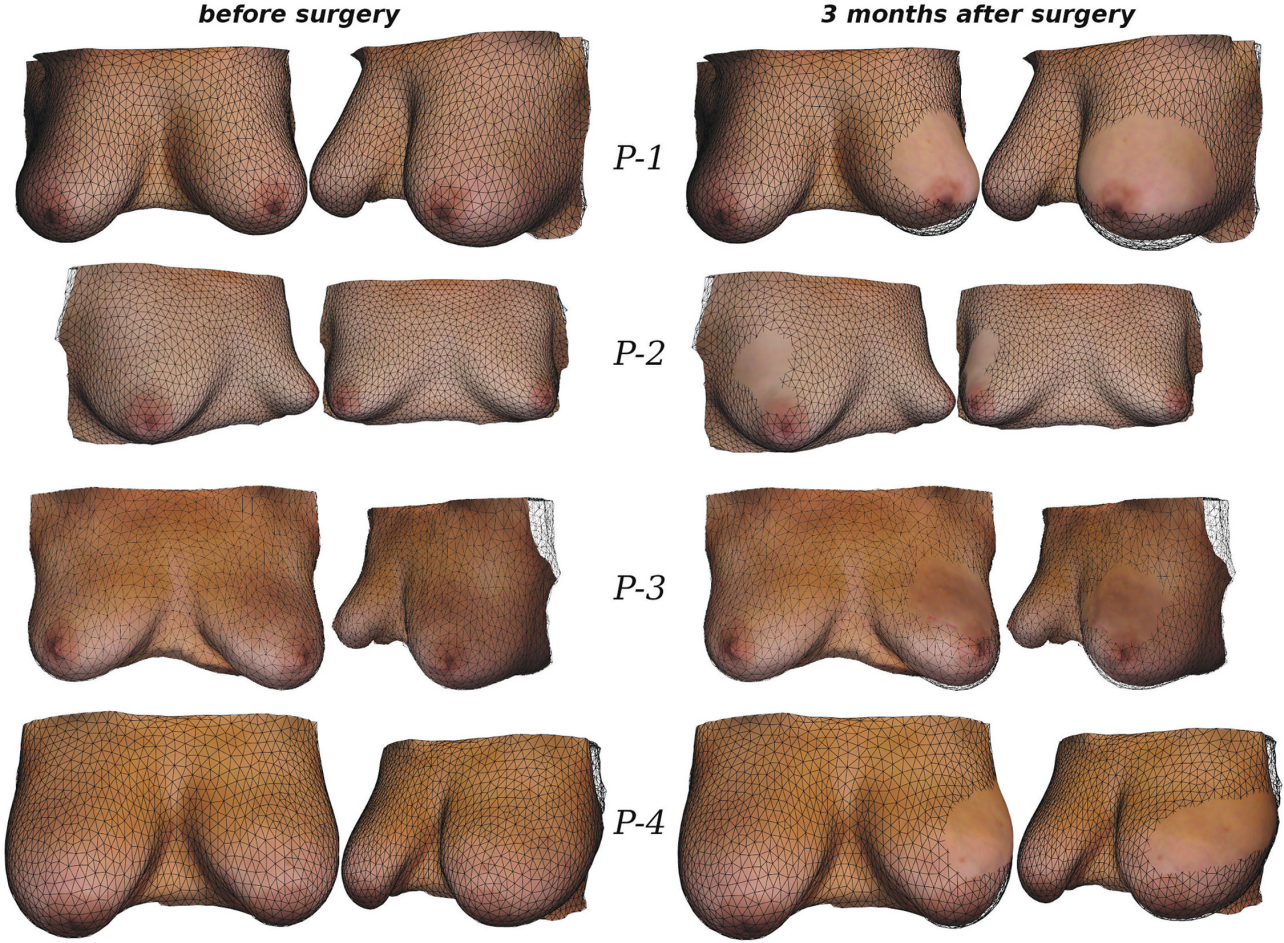
Comparison of simulated breast shape (with texture) before and 3 months after surgery. Baseline biomechanical model (wireframe) with fitted upright surface scan that visualises the skin and oreola texture before surgery (left column). In view of the recorded surgical plan (cf. Fig 4), the surgical simulation tool predicted the breast shape following BCT (right column). The breast deformations resulted from the in-silico analysis are then projected onto the original surface scan to visualise the textured post-surgical breast shape.

To communicate the results between clinical staff and patients, photo-realistic visualisations could be useful to avoid the need for the patient to interpret a non-specific biomechanical mesh. In order to visualise the predicted outcome in a photo-realistic way therefore, we considered the following two issues. First, as reported earlier, the patient’s breast shape can be severely deformed during MRI acquisition. This has been observed in our experiments to have a direct effect on the FE predictions. As a result, skin surface distortion needs to be removed from the breast model. Second, the texture needs to be properly transferred to the patient-specific biomechanical model. To achieve both goals, initially, we rigidly align the optical surface scan of the patient in the upright position with the corresponding upright prediction of the biomechanical model. Subsequently, the residual discrepancy between the simulation and the surface scan is removed by a surface warping technique, resulting in an accurate match of the two meshes involved [47]. This permits not only transfer of the texture onto the skin elements of the biomechanical model but also projection of the deformations predicted by the wound healing/contraction simulation onto the textured skin surface. Fig 8 presents the results of our alignment and displacement projection strategy for all cases, where we compare the textured surface mesh before and after surgery.

**Fig 9:**
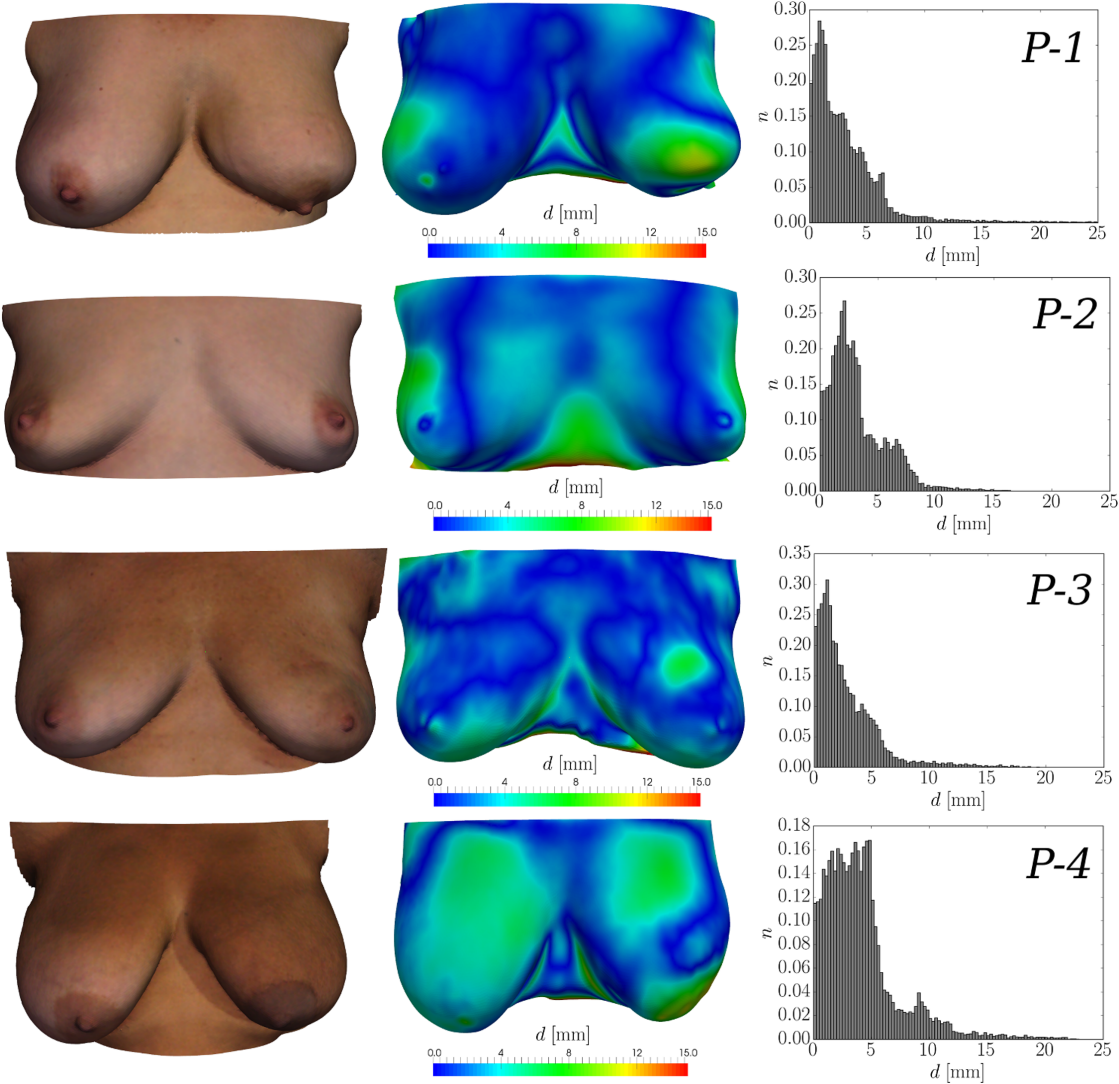
Comparison between the acquired follow-up surface scans and the in-silico predictions. The follow-up surface scan (6–12 months after surgery) with the patients in standing position is illustrated on the left-hand side column of the figure. In the centre column, the breast shape 3 months after BCT—predicted by the surgical simulation tool—is shown. The colour map represents the measured distance, *d*, between the rigidly aligned follow-up scan and the simulated surgical outcome. On the right-hand side column, the histogram to the normalised frequency of occurrence, *n*, against distance, *d*, is presented.

The simulated post-surgical surfaces were further exploited in order to both validate the developed mathematical models, and evaluate the prediction accuracy of the surgical simulation tool. To this end, follow-up data, in the form of 3dMD surface scans, were acquired 6 to 12 months after surgery for each patient and compared directly with the predicted surgical outcome. Rigid alignment between the simulated and acquired follow-up surface is established using an iterative closest point algorithm [48]. After rigid alignment, the surface distance *d* between the simulation and the follow-up acquisition is measured. The results, in terms of mean distance and standard deviation, as well as 50^th^, 90^th^, and 95^th^ percentile values for each patient are given in Table 1. Fig 9 illustrates for each patient case: (i) the follow-up surface scan, (ii) the simulated surgical outcome colour-coded with *d* (in mm), the measured distance between the simulation result and the follow-up surface, and (iii) the corresponding histogram of the distance values.

**Table 1:** Validation results of the in-silico model and the surface optical scans.

Patient	*mean*	*std.*	*50^th^ perc.*	*90^th^ perc.*	*95^th^ perc.*
*P-1*	3.1	3.1	2.3	6.2	8.2
*P-2*	3.2	2.4	2.6	6.7	7.6
*P-3*	2.8	2.7	2.0	5.7	7.7
*P-4*	4.1	3.3	3.5	8.7	10.6

Measurements of the surface distance, *d*, (in mm) between the surgical simulation predictions and the rigidly aligned follow-up optical surface scans. The last three columns give the 50^th^, 90^th^, and 95^th^ percentile values respectively for each patient.

The mean surface distances between the simulation and the follow-up optical surface scan range between 2.8 and 4.1 millimetres. This indicates an excellent simulation accuracy, especially when viewed in the light that additional aspects might have influenced the breast shape during the one-year follow-up period. For *P-2*, for instance, a weight loss between the two image acquisition time points was observed which increased the distance measured between surfaces laterally. The deformity observed for case *P-1* was also well predicted by the simulation in terms of surface displacement. However, the actual deformity was more pronounced than the simulation predicted, more precisely, the change of the orientation of the left nipple from anterior to inferior was not accurately predicted by the simulation. For case *P-3* the simulation predicted a slightly larger influence of surgery in the upper inner quadrant of the left breast than actually occurred. However the overall shape and appearance between simulation and follow-up was very similar. All together, the evaluation with follow-up surface scans suggests that the implemented mechano-biological simulation framework is well capable of predicting surgical outcome with clinically useful accuracy. Slight over- and under-prediction of the actual deformity is to be expected due to inter-patient variability.

In summary, we strongly believe that such photo-realistic illustrations can provide important information to the surgeon and their patient, as well as informing both of the expected deformities occurring at the skin and to the internal structure of the patient’s breast. The proposed computational framework therefore demonstrates significant potential to aid surgical decision making and, hence, improve the accuracy of cosmetic predictions of OBS treatment.

## Discussion

An integrated and validated computational framework for the numerical prediction of oncoplastic breast conserving interventions for early breast-cancer treatment has been presented. The proposed three-dimensional multiscale finite element model is capable of simulating wound healing, angiogenesis and wound contraction of breast tissues. To the authors’ knowledge, this is the first complete three-dimensional, full-scale attempt to realistically model the biomechanics of female breast tissues and the biological processes associated with wound healing and angiogenesis after BCT.

The simulations, derived from routine clinical prone MR scans of four subjects, selected chronologically from a longitudinal cohort of breast conserving surgery patients, have been validated using 3D surface scans acquired six to twelve months after surgery, via a high accuracy 3dMD commercial imaging system. The measured mean surface distances of between 2.8 and 4.1 mm encapsulate errors due to the surgical simulation, as well as other factors including the gross prone to unloaded to upright simulation, population based material parameter selection and external factors such as weight gain or loss.

The present work demonstrates the potential of the oncoplastic breast surgery simulator we have developed to predict the cosmetic outcome of breast conserving therapy. The proposed computational platform could prove a valuable tool for surgical decision support and for multi-disciplinary team meetings, as well as a useful tool to support patient–surgeon interaction in a shared decision making process, to aid reliable aesthetic outcome prediction and assessment.

The present model does not account for the side-effects of adjuvant therapies, e.g. radiotherapy or chemotherapy. Indeed, as Vrieling et al. [49] reported, the final cosmetic outcome of breast conserving interventions is not only dependent on the breast shape, breast size and tumour volume but also on the radiotherapy treatment process itself. As a consequence, it is essential to enhance the current mathematical model to account for the effects of such adjuvant treatment procedures. An outstanding issue is the breast tissue deformation that occurs during the MRI acquisition due, for instance, to skin contact with the scanning device. This can lead to an inaccurate estimation of the gravity-free breast geometry, which will in turn impact the corresponding prediction of the breast shape in the upright pose. For this reason, we are actively investigating methods by which the input MR data could be corrected for any non-gravitational forces present during the image acquisition.

In future work we will also extend the present physiological tissue recovery model to account for pathological healing, by incorporating the effect of radiotherapy treatment into the biomechanics and biochemics modules. We will model the impact of radiotherapy in wound recovery and specifically how radiation alters tissue stiffness and the natural healing mechanism, e.g. changes in the capillary network growth and formation, proliferation of fibroblasts, and varying levels of regulatory growth factors [50]. We will investigate the effects of soft tissue stiffening and fibrosis on the overall breast shape, using non-invasive imaging modalities that can measure elastic properties of tissues, e.g. magnetic resonance elastography [51].

## Supporting Information

S1 File The Biology of Wound Healing and Angiogenesis. (PDF)

S2 File Finite Element Discretisation of the 1st-order PDEs. (PDF)

S3 File Implementation of the Multiscale Mechano-biological FE Framework. (PDF)

S1 Fig Phases of Physiological Wound Healing. Time-history (in days) schematic illustration of the various phases of tissue regeneration and recovery under physiological conditions [7].
(TIF)

S2 Fig MRI Scans of the Breast Cancer Patients. Orthogonal views of magnetic resonance images with the patients lying in prone position.
(TIF)

S3 Fig Patient-specific FE Models. Caudal and lateral views of the three-dimensional finite element meshes of the patient-specific models derived from processing the MRI scans. The tetrahedral elements in the models correspond to fibroglandular tissue while adipose elements are not shown for visualisation purposes.
(TIF)

S1 Table Wound Healing and Angiogenesis Model Parameters. List of all the parameters used in the biochemical finite element breast model, and references to the published papers are also provided.
(PDF)

S2 Table Soft Tissue Biomechanics Model Parameters. List of all the parameters used in the biomechanical finite element breast model, and references to the published papers are also provided.
(PDF)

S3 Table Feature Characteristics Related to Tissue Volume of the Patient-specific Breasts. The model corresponds to the total volume, in cm^3^, of the analysed geometry. In the third column, an estimate of the left (L) and right (R) breast volume is provided, while the fourth and fifth columns indicate the composition of adipose and fibroglandular tissue in each breast respectively. Asterisks denote the operated breast (L or R) where tissues have been “virtually” removed.
(PDF)

S1 Video Wound Healing Simulation for Patient Case *P-4*: Displacement Field. The solid arrows in the animation represent the displacement field that correspond to the breast tissue deformation analysis (from the unloaded to the supine configuration) due to the effect of gravity and the contraction of the wound during the healing process. The colour-map illustrates the magnitude of the displacement vector (in cm). Note also the dynamic change of the initially excised volume of breast tissue which contracts during healing. The two-dimensional plot at the bottom of the animation depicts the dynamic change of the volume of the operated tissue, where the horizontal axis corresponds to days.
(AVI)

S2 Video Wound Healing Simulation for Patient Case *P-4*: Capillary Density. The volume rendering represents the (normalised dimensionless) capillary density, *υ*/*υ*_0_, in the operated tissue region. At the beginning of the simulation no capillaries are present in this region, hence *υ*/*υ*_0_ = 0. During the wound healing simulation new sprouts are forming at the undamaged–operated breast tissue interface, therefore, neo-vascularisation extends (modelled here as a diffusion process) towards the centre of the operated region. At the end of the analysis, after 50 days approximately, the capillary density has been restored locally thus reaching its maximum value, and vascular growth comes nearly to a halt. For explanation of the two-dimensional plot, see the description in S1 Video.
(AVI)

S3 Video Wound Healing Simulation for Patient Case *P-4*: Cell Density. The volume rendering represents the (normalised dimensionless) cell density, *η*/*η*_0_, in the wounded region. At the early stages of the simulation—similarly to S2 Video—cells (i.e. fibroblasts) are migrating and proliferating towards the wound, while mitosis of cells (e.g. myofibroblasts, etc.) is also supported by the newly formed vascular network in this region. Evidently, one month after BCT, an established extracellular matrix is formed, tissue granulation nearly completes and the breast tissue start to remodel and mature, hence, the decreasing rate of deformation at the operated breast. For explanation of the two-dimensional plot, see the description in S1 Video.
(AVI)
