# Supplementary material for: Multiscale Mechano-Biological Finite Element Modelling of Oncoplastic Breast Surgery—Numerical Study towards Surgical Planning and Cosmetic Outcome Prediction
Source: PLoS One. 2016 Jul 28;11(7):e0159766. doi: 10.1371/journal.pone.0159766 (PMC4965022; doi:10.1371/journal.pone.0159766)
Supplement: S1 File — (PDF) [file pone.0159766.s001.pdf]

SUPPORTING INFORMATION

Multiscale Mechano-biological Finite Element Modelling of Oncoplastic Breast Surgery – Numerical Study Towards Surgical Planning and Cosmetic Outcome Prediction

V. Vavourakis, B. Eiben, J.H. Hipwell, N.R. Williams, M. Keshtgar, D.J. Hawkes

The Biology of Wound Healing and Angiogenesis

Wound healing can be divided into several overlapping phases, where three major stages can be identified [1, 2] (see illustration in S1 Fig), namely the inflammatory, the proliferative and the matrix-formation/remodelling phase.

The first stage begins at the time of injury, which in the present study is caused by the incision and excision of breast tissues in the operating theatre, and lasts for 24 to 48 hours. This stage begins with haemostasis (Phase I) and leads to inflammation (Phase II). Platelets form the initial thrombus and release a series of chemical growth factors that induce the chemotaxis and proliferation of neutrophils and macrophages. Platelet derived growth factor (PDGF) is released by platelets in Phase II. It is an important chemical agent that activates and attracts macrophages. Macrophages are the prominent cells in Phase II and are believed to orchestrate tissue regeneration in the wound, coordinating with neutrophils to remove necrotic tissue, debris and bacteria from the wound (phagocytosis). More importantly, they release various growth factors and cytokines that transform the relatively acellular wound into a cellular environment. Pro-inflammatory cytokines and growth factors released by macrophages include the interleukins, PDGF, transforming growth factor-β (TGFβ), epidermal growth factor, fibroblast growth factor (FGF), vascular endothelial growth factor (VEGF) and insulin-like growth factor. In turn, these growth factors attract, recruit, and activate additional macrophages, lymphocytes, fibroblasts and endothelial cells. Amongst the above chemical factors, TGFβ is the major growth factor involved in collagen growth stimulation and synthesis. Subsequently, fibroblasts proliferate to become the dominant cell of the proliferative stage (Phase III), while keratinocytes are also known to epithelialise the wound. Fibroblasts produce collagen which is deposited in the damaged region, hence, providing structure to the wound by replacing the fibronectin-fibrin matrix. It is known that, at the very early stages of healing, fibroblasts produce type-3 collagen (which may account for 30% of the collagen in the wound), while by the second week, collagen of type-1 is mainly produced.

At the same time, genesis of new capillaries occurs to sustain the fibroblast proliferation by providing oxygen and necessary nutrients to the tissue regenerated in the wound. This biological process is known as angiogenesis, or blood vessel growth, and is a critical step in physiological wound healing [3]. During angiogenesis, new capillary sprouts extend to the fibrin-rich wound and within a few days form a microvascular network throughout the granulation tissue; while as collagen accumulates in the granulation tissue to produce a scar, the density of blood vessels diminishes. Moreover, angiogenesis involves the chemotactic interactive response of endothelial cells and a series of angiogenic cytokines produced at the wound site. Chemical proteins that signal angiogenesis include: FGF, VEGF, TGFβ, angiopoietin, and mast cell tryptase. Nonetheless, it has been also reported that wound angiogenesis is regulated by

endothelial cell interaction with the specific three-dimensional extracellular matrix (ECM) environment in the wound space.

Remodelling (Phase IV) is the final stage of wound healing. It begins approximately two to three weeks after injury and can take up to two years. In general, development of normal epithelium and maturation of the scar tissue occurs in Phase IV. At this stage, synthesis and degradation converge to a steady state, as the collagen and other proteins in the wound site progress to an organized cell-matrix layout. Fibroblasts organise and cross-link the collagen, where collagen of type-3 is replaced by type-1 in order to restore the normal dermal collagen composition. Additionally, wound strength gradually increases, contraction of the wound occurs and the wound colour alters as the fibroblast and microvascular network density decreases. Eventually scar tissue regains a structure similar to that of undamaged tissue, but usually the same level of tissue strength is not restored. As the scar matures, the level of vascularity decreases and the scar changes from red through pink to grey with time. However, it is important to note here that all the aforementioned stages of tissue recovery may change in sequence or vary in length because of chronic disease (e.g. diabetes), infection, malnutrition, or other exogenous factors such as radiation.

References

1. Barrientos S, Stojadinovic O, Golinko MS, Brem H, Tomic-Canic M. Growth factors and cytokines in wound healing. Wound Repair and Regeneration. 2008;16(5):585–601.

2. Young A, McNaught CE. The physiology of wound healing. Surgery (Oxford). 2011;29(10):475–479.

3. Tonnesen MG, Feng X, Clark RAF. Angiogenesis in Wound Healing. Journal of Investigative Dermatology Symposium Proceedings. 2000;5(1):40–46.
