# Supplementary material for: Multiscale Mechano-Biological Finite Element Modelling of Oncoplastic Breast Surgery—Numerical Study towards Surgical Planning and Cosmetic Outcome Prediction
Source: PLoS One. 2016 Jul 28;11(7):e0159766. doi: 10.1371/journal.pone.0159766 (PMC4965022; doi:10.1371/journal.pone.0159766)
Supplement: S2 File — (PDF) [file pone.0159766.s002.pdf]

Multiscale Mechano-biological Finite Element Modelling of Oncoplastic Breast Surgery – Numerical Study Towards Surgical Planning and Cosmetic Outcome Prediction

V. Vavourakis, B. Eiben, J.H. Hipwell, N.R. Williams, M. Keshtgar, D.J. Hawkes

Finite Element Discretisation of the 1st-order PDEs

Assume that the analysed domain occupies volume Ω and it is bounded by the boundary Γ. Taking a proper weighted function space for all five species, the variational principle of equation 7 in the manuscript, after making use of Gauss's divergence theorem, reads

$$\int_\Omega \delta\mathbf{p}^T \dot{\mathbf{q}}\, dV = \int_\Omega \delta\mathbf{p}^T \mathbf{S}\, dV - \int_\Omega \boldsymbol{\nabla}\delta\mathbf{p} : [\mathbf{D}\cdot\boldsymbol{\nabla}\mathbf{q}]\, dV + \int_\Gamma \delta\mathbf{p}^T [\mathbf{D}\cdot\boldsymbol{\nabla}\mathbf{q}]\cdot\hat{\mathbf{n}}\, dS\,, \tag{1}$$

where $\hat{\mathbf{n}}$ denotes the outward unit vector to Γ, and $\delta\mathbf{p}$ the variation of the weighted variables. Now, assuming that the flux of the species is zero on the boundary of the analysed breast geometry, the rightmost term in the above integral equation vanishes.

Introducing linear finite elements and using the same Lagrange polynomial basis (and of the same order) for all species, one can obtain the final system of the PDEs involved in the wound healing model:

$$\mathbf{M}\cdot\dot{\mathbf{q}} = \mathbf{F}\,, \tag{2}$$

where the "mass" matrix is given by

$$\mathbf{M}^{(i,j)} = \begin{bmatrix} \ddots & \dots & 0 \\ \vdots & m^{(i,j)} & \vdots \\ 0 & \dots & \ddots \end{bmatrix}, \quad m^{(i,j)} = \int_{\Omega_e} \Phi^{(i)}\,\Phi^{(j)}\, dV\,, \tag{3}$$

and the right-hand-side "force" vector

$$\mathbf{F}^{(i)} = \left\{ \begin{array}{c} \int_{\Omega_e} \left\{ \Phi^{(i)} \left[K\,S\,(2\,\eta_0\,\eta - \eta^2) - k\,\eta \right] - D_\eta\, \boldsymbol{\nabla}\Phi^{(i)}\cdot\boldsymbol{\nabla}\eta \right\} dV \\ \int_{\Omega_e} \left\{ \Phi^{(i)}\, (\ell_\eta\,\eta - \ell\,\varsigma) - D_\varsigma\, \boldsymbol{\nabla}\Phi^{(i)}\cdot\boldsymbol{\nabla}\varsigma \right\} dV \\ \int_{\Omega_e} \Phi^{(i)} \left[\beta\, H\,(\upsilon_0\,\upsilon - \upsilon^2) \right] dV \\ \int_{\Omega_e} \left\{ \Phi^{(i)}\, (\lambda_\upsilon\,\upsilon - \lambda\,\xi) - D_\xi\, \boldsymbol{\nabla}\Phi^{(i)}\cdot\boldsymbol{\nabla}\xi \right\} dV \\ \int_{\Omega_e} \left\{ \Phi^{(i)}\, (\phi_\xi\, Q - \phi\,\mu) - D_\mu\, \boldsymbol{\nabla}\Phi^{(i)}\cdot\boldsymbol{\nabla}\mu \right\} dV \end{array} \right\}, \tag{4}$$

where the Latin indices correspond to the nodes associated to each finite element "e" (having volume Ω_e) where the assembly takes place. The i-node shape functions are denoted with $\Phi^{(i)}$ and $\boldsymbol{\nabla}\Phi^{(i)}$ the gradients.

Nonlinear equation 2 can be integrated using an explicit or an implicit time-integration algorithm. In the present numerical framework, we choose to utilise the

former scheme where a lumped mass matrix (e.g. using the row-sum method) is considered to substantially increase the computational speed and reduce memory usage. However, detailed description of the numerical implementation of the explicit time integration scheme for first-order differential equations can be found in the FE textbook of Bathe [1] and Zienkiewicz et al. [2].

References

1. Bathe KJ. Finite Element Procedures. Prentice Hall; 1996.

2. Zienkiewicz OC, Taylor RL, Nithiarasu P. The Finite Element Method for Fluid Dynamics. 6th ed. Butterworth-Heinemann; 2005.
