# Supplementary material for: Multiscale Mechano-Biological Finite Element Modelling of Oncoplastic Breast Surgery—Numerical Study towards Surgical Planning and Cosmetic Outcome Prediction
Source: PLoS One. 2016 Jul 28;11(7):e0159766. doi: 10.1371/journal.pone.0159766 (PMC4965022; doi:10.1371/journal.pone.0159766)
Supplement: S3 File — (PDF) [file pone.0159766.s003.pdf]

## SUPPORTING INFORMATION

# Multiscale Mechano-biological Finite Element Modelling of Oncoplastic Breast Surgery – Numerical Study Towards Surgical Planning and Cosmetic Outcome Prediction

V. Vavourakis, B. Eiben, J.H. Hipwell, N.R. Williams, M. Keshtgar, D.J. Hawkes

## Implementation of the Multiscale Mechano-biological FE Framework

The proposed multiscale mechano-biological Finite Element (FE) framework has been incorporated into the existing in-house numerical analysis framework *FEB3* – pronounced *Phoebe* – which is freely available upon request from: https://bitbucket.org/vasvav/feb3-finite-element-bioengineering-in-3d/wiki/Home. The FE computational framework has been implemented in an object-oriented, scalable, C++ code that incorporates several open-source numerical libraries:

- *blitz++*[1] is an meta-template library in C++ which was utilised in *FEB3* for tensor algebra and multi-dimensional tensor manipulation [1].
- *GNU Scientific Library* is an ANSI-C library that contains an wide range of mathematical routines (over 1000 in total) such as random number generators, special functions, statistics, numerical differentiation, data fitting, etc. (see online documentation: https://www.gnu.org/software/gsl/manual/html_node/) [2].
- *METIS*[2] and *ParMETIS*[3] is a pair of libraries containing established algorithms for partitioning graphs, partitioning finite element meshes and producing fill-reducing orderings for sparse matrices, in serial and in parallel computing respectively [3].
- *MPICH*[4] is a standardized and portable message-passing system which is a communication protocol for programming parallel computers.
- *PETSc*[5] is a suite of data structures and routines for the solution of scientific applications, and is been used within *FEB3* in solving linear and nonlinear systems [4, 5]. *PETSc* is also integrated with *MPICH* to facilitate parallel computations, while it communicates with *METIS* and *ParMETIS* for sparse system partitioning.
- *libMesh*[6] [6] is the top-level library used by *FEB3*. *libMesh* is an object-oriented C++ framework for the numerical simulation of partial differential equations using arbitrary unstructured discretisations on serial and parallel platforms, while it integrates with high-performance computing libraries such as *PETSc*.

[1] http://sourceforge.net/projects/blitz/
[2] http://glaros.dtc.umn.edu/gkhome/metis/metis/overview
[3] http://glaros.dtc.umn.edu/gkhome/metis/parmetis/overview
[4] www.mpich.org
[5] http://www.mcs.anl.gov/petsc/
[6] http://libmesh.github.io/

## References


1. Veldhuizen T. Blitz++ User's Guide: A C++ class library for scientific computing; 2006. Available from: `http://blitz.sourceforge.net/resources/blitz-0.9.pdf`.

2. Galassi M, Davies J, Thailer J, Gough B, Jungman G, Alken P, et al.. GNU Scientific Library Reference Manual; 2013. Available from: `http://www.gnu.org/software/gsl/`.

3. Karypis G, Kumar V. MeTis: Unstructured Graph Partitioning and Sparse Matrix Ordering System, Version 4.0; 2009. `http://www.cs.umn.edu/~metis`.

4. Balay S, Gropp WD, McInnes LC, Smith BF. Efficient Management of Parallelism in Object Oriented Numerical Software Libraries. In: Arge E, Bruaset AM, Langtangen HP, editors. Modern Software Tools in Scientific Computing. Birkhäuser Press; 1997. p. 163–202.

5. Balay S, Abhyankar S, Adams MF, Brown J, Brune P, Buschelman K, et al. PETSc Users Manual. Argonne National Laboratory; 2014. ANL-95/11 - Revision 3.5. Available from: `http://www.mcs.anl.gov/petsc`.

6. Kirk BS, Peterson JW, Stogner RH, Carey GF. `libMesh`: A C++ Library for Parallel Adaptive Mesh Refinement/Coarsening Simulations. Engineering with Computers. 2006;22(3–4):237–254.
