# Supplementary figures and images for: Multiscale Mechano-Biological Finite Element Modelling of Oncoplastic Breast Surgery—Numerical Study towards Surgical Planning and Cosmetic Outcome Prediction
Source: PLoS One. 2016 Jul 28;11(7):e0159766. doi: 10.1371/journal.pone.0159766 (PMC4965022; doi:10.1371/journal.pone.0159766)

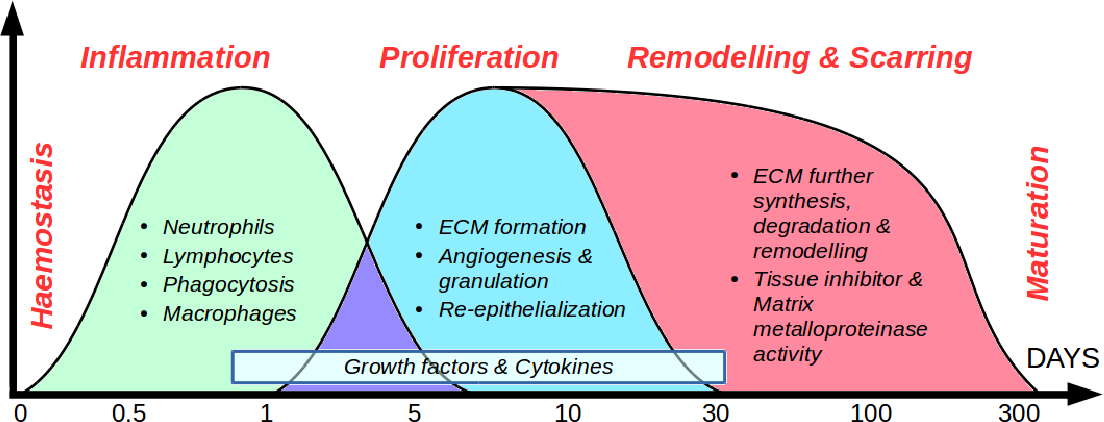

Supplement: S1 Fig — Time-history (in days) schematic illustration of the various phases of tissue regeneration and recovery under physiological conditions [7]. (TIF) [file pone.0159766.s004.tif]

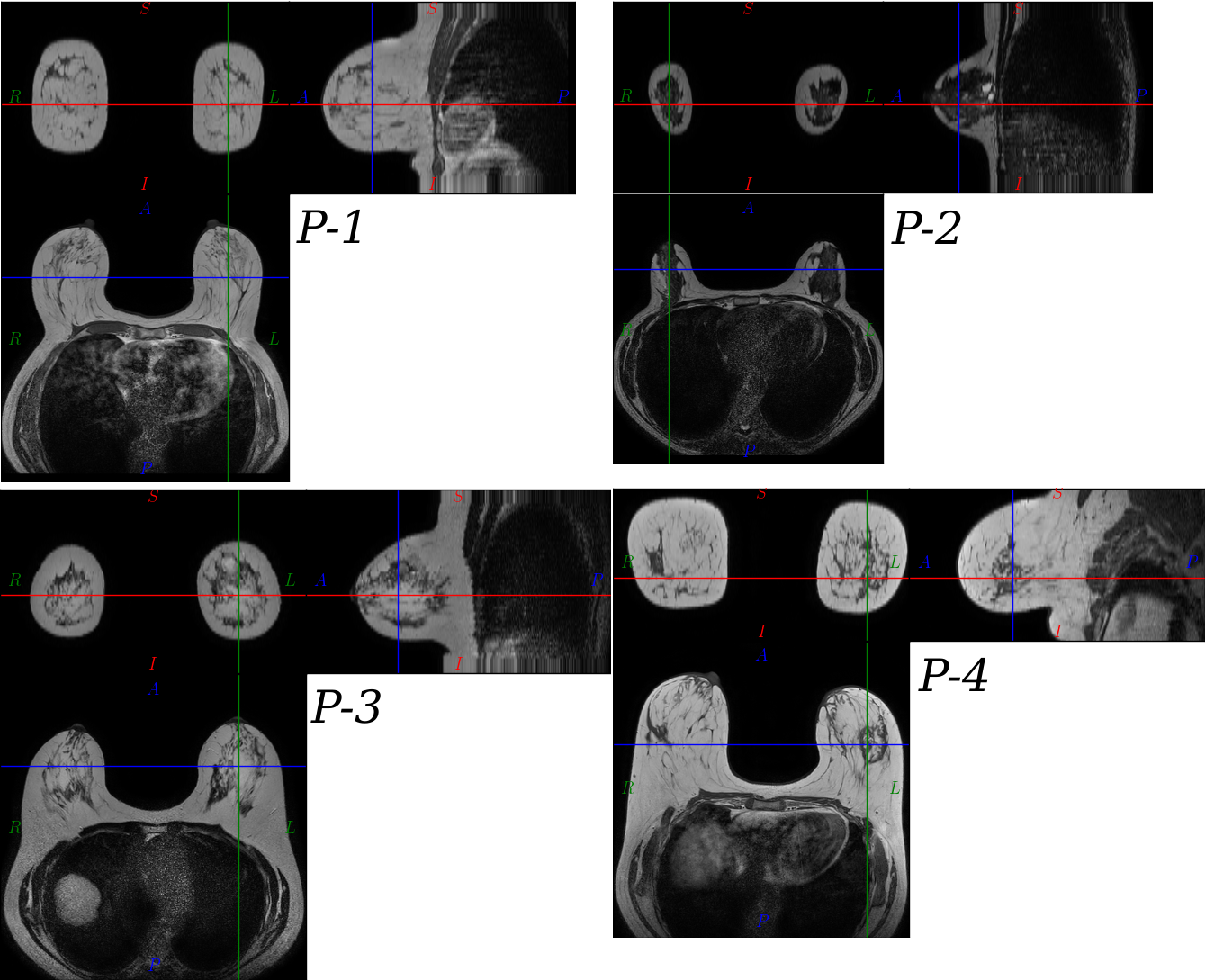

Supplement: S2 Fig — Orthogonal views of magnetic resonance images with the patients lying in prone position. (TIF) [file pone.0159766.s005.tif]

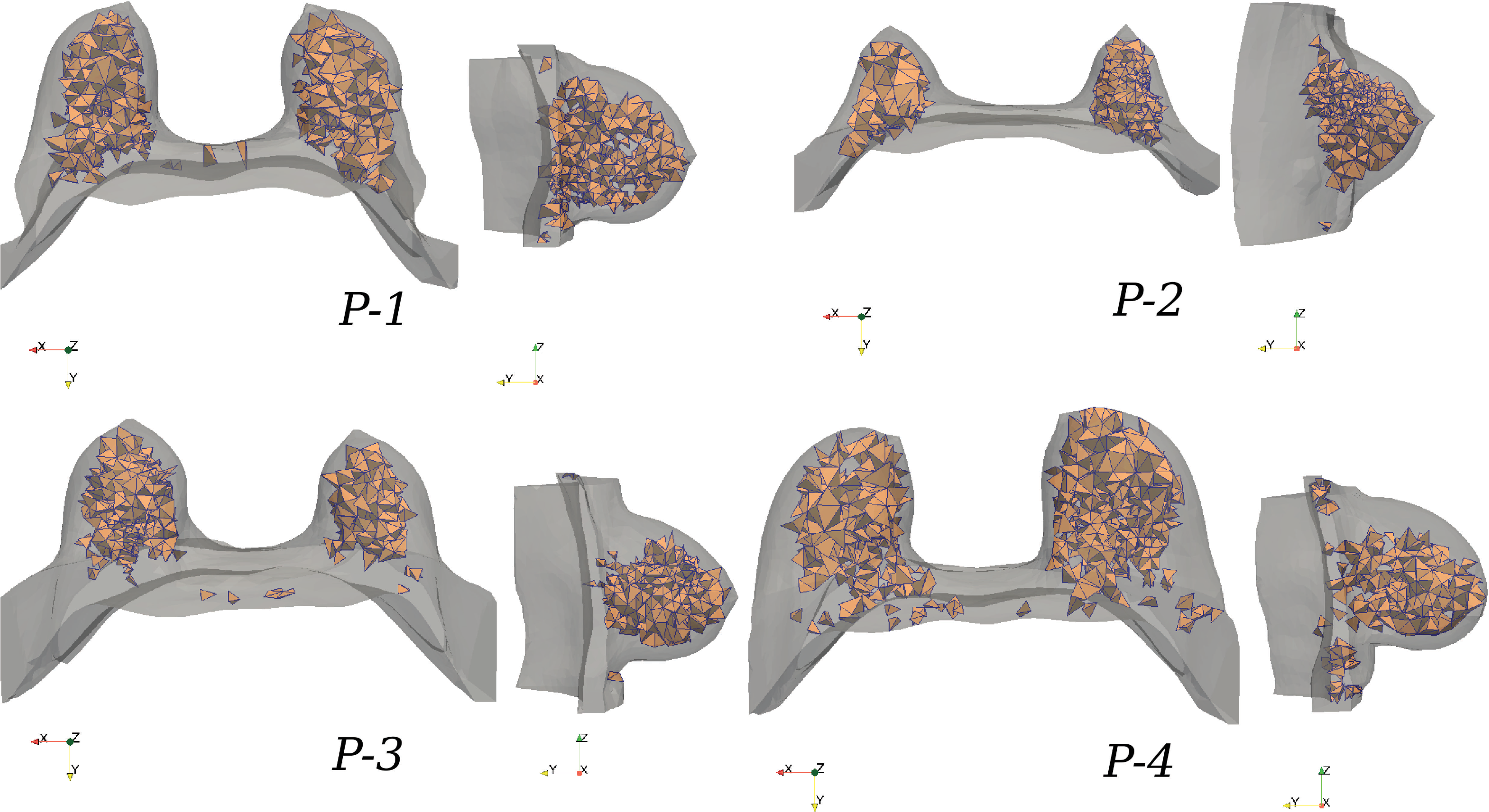

Supplement: S3 Fig — Caudal and lateral views of the three-dimensional finite element meshes of the patient-specific models derived from processing the MRI scans. The tetrahedral elements in the models correspond to fibroglandular tissue while adipose elements are not shown for visualisation purposes. (TIF) [file pone.0159766.s006.tif]
