# Supplementary material for: Multiscale Mechano-Biological Finite Element Modelling of Oncoplastic Breast Surgery—Numerical Study towards Surgical Planning and Cosmetic Outcome Prediction
Source: PLoS One. 2016 Jul 28;11(7):e0159766. doi: 10.1371/journal.pone.0159766 (PMC4965022; doi:10.1371/journal.pone.0159766)
Supplement: S1 Table — List of all the parameters used in the biochemical finite element breast model, and references to the published papers are also provided. (PDF) [file pone.0159766.s007.pdf]

SUPPORTING INFORMATION

Multiscale Mechano-biological Finite Element Modelling of Oncoplastic Breast Surgery – Numerical Study Towards Surgical Planning and Cosmetic Outcome Prediction

V. Vavourakis, B. Eiben, J.H. Hipwell, N.R. Williams, M. Keshtgar, D.J. Hawkes

Wound Healing and Angiogenesis Model Parameters

List of all the parameters used in the biochemical finite element breast model, and references to the published papers are also provided.

**Parameter**	**Value**	**Source**
D_η	5.962×10^{-6} cm^2 d^{-1}	[1]
η_0	$9. \times 10^8$ cell cm^{-3}	[2]
k_ς	2.16×10^8 cm^3 cell^{-1} d^{-1}	Adapted from [1]
k	0.24 d^{-1}	[1]
D_ς	50.976×10^{-3} cm^2 d^{-1}	[1]
ς_0	10^{-8} g cm^{-3}	Adapted from [3]
ℓ_η	1.33×10^{-16} g cell^{-1}d^{-1}	[1]
ℓ	1.20 d^{-1}	[1]
$\bar{\varsigma}$	$4. \times 10^{-7}$ g cm^{-3}	[1]
h_0	10. –	[1]
h_1; h_2	9.47 –; 0.53 –	Adapted from [1]
υ_0	70. cm^{-1}	[4]
β	2.857×10^{-3} cm d^{-1}	Adapted from [1]
D_ξ	4.32×10^{-3} cm^2 d^{-1}	[3]
λ_υ	1.143×10^{-2} cm d^{-1}	Adapted from [5]
λ	0.8 d^{-1}	Adapted from [5]
D_μ	8.64×10^{-2} cm^2 d^{-1}	[3]
μ_0	10^{-8} g cm^{-3}	[6]
ϕ_ξ	10^{-8} g cm^{-3}d^{-1}	Adapted from [3]
ϕ	0.95 d^{-1}	Adapted from [3]
$\hat{\xi}$	0.5 –	[7]
$\bar{\xi}$	0.8 –	Estimated

References

1. Sherratt JA, Murray JD. Mathematical analysis of a basic model for epidermal wound healing. Journal of Mathematical Biology. 1991;29(5):389–404.
2. Tsuji W, Rubin JP, Marra KG. Adipose-derived stem cells: Implications in tissue regeneration. World Journal of Stem Cells. 2014;6(3):312–321.
3. Valero C, Javierre E, García-Aznar JM, Gómez-Benito MJ. Numerical modelling of the angiogenesis process in wound contraction. Biomechanics and Modeling in Mechanobiology. 2013;12(2):349–360.
4. Hilmas DE, Gillette EL. Morphometric analyses of the microvasculature of tumors during growth and after X-irradiation. Cancer. 1974;33(1):103–110.
5. Javierre E, Moreo P, Doblaré M, García-Aznar JM. Numerical modeling of a mechano-chemical theory for wound contraction analysis. International Journal of Solids and Structures. 2009;46(20):3597–3606.
6. Olsen L, Sherratt JA, Maini PK. A Mechanochemical Model for Adult Dermal Wound Contraction and the Permanence of the Contracted Tissue Displacement Profile. Journal of Theoretical Biology. 1995;177(2):113–128.
7. Xue C, Friedman A, Sen CK. A mathematical model of ischemic cutaneous wounds. Proceedings of the National Academy of Sciences. 2009;106(39):16782–16787.
