# Supplementary material for: Multiscale Mechano-Biological Finite Element Modelling of Oncoplastic Breast Surgery—Numerical Study towards Surgical Planning and Cosmetic Outcome Prediction
Source: PLoS One. 2016 Jul 28;11(7):e0159766. doi: 10.1371/journal.pone.0159766 (PMC4965022; doi:10.1371/journal.pone.0159766)
Supplement: S2 Table — List of all the parameters used in the biomechanical finite element breast model, and references to the published papers are also provided. (PDF) [file pone.0159766.s008.pdf]

SUPPORTING INFORMATION

Multiscale Mechano-biological Finite Element Modelling of Oncoplastic Breast Surgery – Numerical Study Towards Surgical Planning and Cosmetic Outcome Prediction

V. Vavourakis, B. Eiben, J.H. Hipwell, N.R. Williams, M. Keshtgar, D.J. Hawkes

Soft Tissue Biomechanics Model Parameters

List of all the parameters used in the biomechanical finite element breast model, and references to the published papers are also provided.

**Parameter**	**Value**	**Source**
$c_{1,\text{adipose}}$; $c_{2,\text{adipose}}$	80. Pa; 0. Pa	[1, 2]
$c_{1,\text{fibrogland}}$; $c_{2,\text{fibrogland}}$	120. Pa; 0. Pa	[1, 2]
$c_{1,\text{recover}}$; $c_{2,\text{recover}}$	300. Pa; 0. Pa	Estimated
κ	20. kPa	Incompressibility Parameter
$\rho_{0,\text{adipose}}$	910. kg m^{-3}	[3]
$\rho_{0,\text{fibrogland}}$	1020. kg m^{-3}	Adapted from [4]
$\rho_{0,\text{recover}}$	1000. kg m^{-3}	Estimated
α_s; β_s	92.4 Pa; 4.4 –	[5]
$c_{2,\text{skin}}$	-203.4 Pa	[5]
$\rho_{0,\text{skin}}$	1060. kg m^{-3}	[5]
τ_f	1.25×10^{-5} N cm cell^{-1}	Adapted from [6]
α_f	1. –	Adapted from [7]
$\eta_{0,\text{f}}$	10^4 cell cm^{-3}	[6]

References

1. del Palomar AP, Calvo B, Herrero J, López J, Doblaré M. A finite element model to accurately predict real deformations of the breast. Medical Engineering & Physics. 2008;30(9):1089–1097.

2. Tanner C, Schnabel JA, Hill DLG, Hawkes DJ, Leach MO, Hose DR. Factors influencing the accuracy of biomechanical breast models. Medical Physics. 2006;33(6):1758–1769.

3. Farvid MS, Ng TW, Chan DC, Barret PH, Watts GF. Association of adiponectin and resistin with adipose tissue compartments, insulin resistance and dyslipidaemia. Diabetes, Obesity & Metabolism. 2005;7(4):406–413.

4. Han L, Hipwell JH, Tanner C, Taylor Z, Mertzanidou T, Cardoso J, et al. Development of patient-specific biomechanical models for predicting large breast deformation. Physics in Medicine and Biology. 2012;57(2):455–472.

5. Veronda DR, Westmann RA. Mechanical characterization of skin – Finite deformations. Journal of Biomechanics. 1970;3(1):111–124.

6. Olsen L, Sherratt JA, Maini PK. A Mechanochemical Model for Adult Dermal Wound Contraction and the Permanence of the Contracted Tissue Displacement Profile. Journal of Theoretical Biology. 1995;177(2):113–128.

7. Javierre E, Moreo P, Doblaré M, García-Aznar JM. Numerical modeling of a mechano-chemical theory for wound contraction analysis. International Journal of Solids and Structures. 2009;46(20):3597–3606.
