# Supplementary material for: Multiscale Mechano-Biological Finite Element Modelling of Oncoplastic Breast Surgery—Numerical Study towards Surgical Planning and Cosmetic Outcome Prediction
Source: PLoS One. 2016 Jul 28;11(7):e0159766. doi: 10.1371/journal.pone.0159766 (PMC4965022; doi:10.1371/journal.pone.0159766)
Supplement: S3 Table — The model corresponds to the total volume, in cm3, of the analysed geometry. In the third column, an estimate of the left (L) and right (R) breast volume is provided, while the fourth and fifth columns indicate the composition of adipose and fibroglandular tissue in each breast respectively. Asterisks denote the operated breast (L or R) where tissues have been “virtually” removed. (PDF) [file pone.0159766.s009.pdf]

# SUPPORTING INFORMATION

# Multiscale Mechano-biological Finite Element Modelling of Oncoplastic Breast Surgery – Numerical Study Towards Surgical Planning and Cosmetic Outcome Prediction

V. Vavourakis, B. Eiben, J.H. Hipwell, N.R. Williams, M. Keshtgar, D.J. Hawkes

## Feature Characteristics Related to Tissue Volume of the Patient-specific Breasts

The model corresponds to the total volume, in $cm^3$, of the analysed geometry. In the third column, an estimate of the left (L) and right (R) breast volume is provided, while the fourth and fifth columns indicate the composition of adipose and fibroglandular tissue in each breast respectively. Asterisks denote the operated breast (L or R) where tissues have been "virtually" removed.

| **Patient** | **Model** | **Breast (L/R)** | **Adipose (L/R)** | **Fibrogland. (L/R)** | **Excision** |
|---|---|---|---|---|---|
| *P-1* | 2733.0 | 1053.7*/1093.6 | 895.4/932.7 | 158.3/160.9 | 21.3 |
| *P-2* | 1050.8 | 294.7/264.4* | 212.9/193.3 | 81.8/71.0 | 5.3 |
| *P-3* | 3556.6 | 1157.7*/1024.9 | 1001.5/917.3 | 156.2/107.7 | 24.8 |
| *P-4* | 4035.5 | 1232.5*/1557.7 | 1022.3/1328.8 | 210.2/228.8 | 63.0 |
